# A Universal Structural Grammar in Enzyme Fold for Predicting Drug Target Stability: Deciphering Directional Scaffolding via Multi-Stage Pearson Correlation of Asymmetric Contact Matrices

**DOI:** 10.3390/pharmaceutics18060728

**Published:** 2026-06-12

**Authors:** Fatin Jannus, Hilario Ramírez-Rodrigo

**Affiliations:** Department of Biochemistry and Molecular Biology I, Faculty of Sciences, University of Granada, Av. Fuentenueva, 18071 Granada, Spain

**Keywords:** structural determinism, stability landscape, hydrolase, Pearson correlation, directional rules, structure-based drug design

## Abstract

**Background/Objectives:** Traditional protein contact analysis often fails to distinguish between local, sequence-driven motifs and global, tertiary scaffolding, which ensures structural determinism. While deep-learning models do not fully elucidate the ‘why’, they do reveal the underlying directional rules of the stability landscape. In this study, we analyzed 475 non-redundant Protein Data Bank (PDB) structures categorized into SCOP classes (all-α, all-β, α/β, α+β) of the hydrolase superfamily. **Methods:** To isolate the structural anchors of the global fold, we applied a sequence separation filter of ∣i − j∣ ≥ 6 and a precise spatial cutoff of 3–5 Å between Cα-only to construct asymmetric 20 × 20 frequency matrices, both raw and normalized, then present the former using a violin diagram. We developed a Pearson Correlation (PC) framework to analyze these matrices, providing high correlation when considered as vectors and giving the directionality (N-to-C vs. C-to-N) in protein folding when considered as matrices. **Results:** Our results reveal a hierarchical organization of tertiary determinism. Initial visualization of Residue–Residue Contact Frequency Matrices (RRCFMs), Z-score normalization (NRRCFM), and violin plots reveal the Universal Structural Grammar (USG) of interaction. Furthermore, a near-perfect PC (r = 0.99) as determined via inter-class Z-score correlation and inter-class PC demonstrates shared statistical interaction laws. In addition, PC Stage 1 (intra-class) analysis identified high symmetry, with around 80% of contacts exhibiting a very strong to strong positive correlations, while PC Stage 2 (inter-class) analysis demonstrated that around 50% of contacts exhibited very strong to strong positive correlations. Finally, we identified universal druggable pockets for drug discovery. **Conclusions:** This powerful mathematical framework provides a robust analytical tool for structure-based drug design.

## 1. Introduction

Predicting residue–residue contacts is essential for understanding protein stability, de novo design, and the binding affinity of therapeutic molecules [1,2]. Over the past several decades, bioinformatic approaches have significantly improved our ability to predict protein structural classes [3,4,5,6,7,8,9,10,11,12,13,14]. Recently, AlphaFold has transformed the field by achieving unprecedented accuracy in predicting 3D coordinates from primary sequences [15]. However, a fundamental challenge remains: while AlphaFold successfully provides the ‘what’ (the final geometric coordinates), the ‘why’ the underlying structural grammar and the physical logic that govern global stability remains a black box [16,17].

The stability of a protein is not merely a product of its local secondary structure, but rather the result of a complex, long-range network of non-covalent interactions. Most existing contact map analyses focus heavily on local sequence neighbors, which often masks the true chemical glue that maintains the global fold [18,19,20].

The structural integrity and functional dynamics of a protein fold are dictated by the spatial arrangement of the protein’s amino acids. To bypass the prohibitive computational costs of full atomistic simulations, Residue Interaction Networks (RINs) have emerged as highly effective topological paradigm [21,22]. While comprehensive frameworks such as the Protein Contacts Atlas (PCA) demonstrate that all-atom networks govern protein stability and allosteric signaling, traditional models often employ broad spatial thresholds that introduce significant topological noise. To overcome these limitations, this work condenses these structural principles into a simplified, coarse-grained framework focusing exclusively on carbon-alpha (C_α_) coordinates [23].

Validating contacts within a strict, open spatial interval between 3.0 Å and 5.0 Å ensures the precise capture of immediate backbone scaffolding constraints such as local helical pitches and adjacent strand alignments while preventing the network from becoming overly connected and uninformative [23,24]. Furthermore, combining this spatial cutoff with a strict sequence-separation filter (∣i − j∣ ≥ 6) focuses solely on long-range tertiary determinants, effectively isolating high-fidelity distal tertiary contacts from local conformational redundancies. Through this dual filtering process, we isolate the high-fidelity distal backbone contacts, or structural anchors, that constitute the protein’s core scaffolding. Additionally, this topological framework maps the underlying geometrical environment that accommodates critical structural macro-arrangements, maintaining the stability required for protein target characterization and modern drug discovery pipelines [25,26,27,28,29]. Understanding these distal networks is essential for advancing protein design and solving the inverse folding problem, both of which have been widely studied in previous research [30,31,32].

This structural understanding is particularly important for the hydrolase superfamily, which is the largest and most diverse class of enzymes. Hydrolases, such as serine hydrolase, α/β-hydrolase domain 6 (ABHD6), and PTPRK (protein tyrosine phosphatase receptor type K), are key targets in the treatment of conditions including Alzheimer’s disease, obesity, type 2 diabetes, and cancer [33,34,35,36,37]. In our previous research, we employed traditional drug discovery methods to identify the pharmacological properties of various compounds [38,39,40,41]. We also evaluated protein–protein interaction networks to identify the genes and biological processes that are most significantly involved in channelopathies [42]. Improving drug discovery and design would therefore enable us to improve the treatment of several diseases. As the effectiveness of drug design is greatly influenced by the structural class of the targets in question, identifying the underlying contact grammar of their respective folds is essential. Crucially, our research provides statistical proof for inherent directionality in protein fold prediction. While previous studies have suggested directional preferences [43,44], our analysis of asymmetric frequency matrices formally demonstrates that the N-to-C versus C-to-N orientation of residue packing is a deterministic feature of fold architecture. Several studies have previously investigated the fractal law in protein structure [45,46,47,48]. Our research provides a new perspective on the fractal nature of protein stability, as revealed by the PC framework, which is highly correlated with hydrophobic, polar, and charged interactions forming binding sites, as previously proven [49,50,51,52]. The PC approach has also been used by previous studies to improve the prediction of protein structure [53,54,55].

In this study, we move beyond simple binary contact maps to develop class-specific Residue–Residue Contact Frequency Vectors (RRCFVs) and Matrices (RRCFMs + NRRCFMs). By shifting the focus from simple spatial coordinates to the directional correlation of long-range residues, this analysis provides a systematic characterization of protein structural grammar. Using a PC framework, we analyze 475 non-redundant hydrolase structures categorized into four fundamental classes: all-α (118 PDB), all-β (114 PDB), α/β (117 PDB), and α+β (126 PDB), corresponding to SCOP classifications [56]. We demonstrate that the RRCFM, NRRCFM, and RRCFV appear approximately similar across divergent classes, suggesting that there is a USG of interaction with an underlying directional logic that contains the specific fingerprints required for predicting the structural stability of the drug target.

Based on our systematic analysis of RRCF, we propose the following hypotheses regarding the existence of a USG in hydrolase proteins:

1. Conservation of interaction hierarchy: We hypothesize that the top-ranked hydrophobic contacts will maintain a Pearson correlation threshold of r > 0.99 across all four SCOP classes, indicating that the core of the USG is invariant to global fold topology.
2. Directional bias invariance: We predict that the asymmetry observed in contact matrices reflecting N-to-C terminal directionality will show consistent divergence when comparing upper and lower triangles, suggesting that the directional vector of folding is a fundamental rule of the USG.
3. Geometric saturation threshold: We hypothesize that, as protein length increases, the local contact density within the 3–5 Å range will converge to a constant value, proving that the USG operates under strict geometric saturation constraints regardless of total protein volume.

Our goal in testing these hypotheses is to formalize the rules that govern hydrolase protein architecture and provide an analytical framework for structural biology. We will then map the universal stability constants of the proteome, thereby providing a mathematical foundation for improving target reliability and reducing off-target risks in drug design.

## 2. Materials and Methods

**Figure 1:**
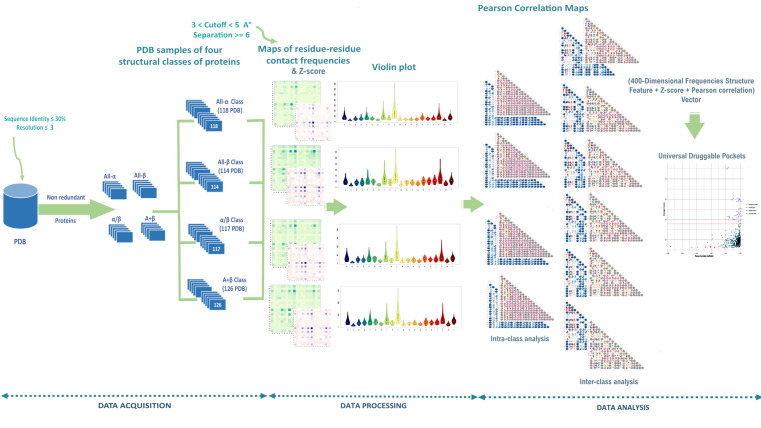
An overview of the workflow containing three major stages: 1. data acquisition, 2. data processing, 3. data analysis.

Figure 1 presents an overview of the workflow of the proposed method. It consists of the following steps: data acquisition, data processing, and data analysis. The details of each process are described below.

### 2.1. Data Acquisition

#### Databases

From the Protein Data Bank (PDB) (www.rcsb.org), we selected 475 hydrolase protein structures from the all-α, all-β, α+β, and α/β classes. The set contained proteins with no more than 30% pairwise sequence identity, to avoid redundancy and evolutionary bias. The proteins structures were solved by X-ray diffraction, with a resolution equal to 3 Å or lower, and we considered only the first subunit. Contact site frequency was examined in non-redundant structural classes of hydrolase proteins categorized by the SCOP database. Proteins were classified with SCOP nomenclature into four classes, all-α, all-β, α+β, and α/β, with 118, 114, 117, and 126 proteins, respectively (Supplementary Figure S1).

### 2.2. Data Processing

#### 2.2.1. Contact Site Variables

The occurrence pattern of contact sites in proteins is defined as follows: the distance between the carbon-alpha (Cα-only) of two different amino acids is not greater than a specified distance value (cutoff), and the contacting amino acids in a protein sequence are separated by not less than a specified number of amino acids (separation). In our study, two neighboring residues in a sequence have a separation value of 6 amino acids or higher. We found contact sites using cutoff values between 3 and 5 Å.

#### 2.2.2. Tertiary Interaction Constraint

To isolate tertiary stability from local secondary motifs, a sequence separation filter was applied, and only residue pairs (i, j) satisfying the condition were retained. We defined the contact frequency of each specified residue–residue contact. The contact frequency of all amino acids in the analyzed proteins was assessed for the previous cutoff and separation values. Contact frequencies were analyzed separately for the all-α, all-β, α+β, and α/β classes.

#### 2.2.3. Residue–Residue Contact

We considered residue **A_i_** to be in contact with residue **A_j_** if it is inside our definition of contact. Then, the frequency contact **A_i_A_j_** = 1, while on the contrary **A_i_A**j = 0. The amino acid composition of a protein is expressed as a 20 × 20 matrix of A, C, D, E, F, G, H, I, K, L, M, N, P, Q, R, S, T, V, W, and Y, where each letter corresponds to a single amino acid letter code. For each class, 20 × 20 asymmetric frequency matrices were constructed. Unlike symmetric contact maps, these matrices preserved the sequential order of the residue, where the row (residue **i**) presents the residue that appears first in the primary sequence, and the column (residue **j**) presents the residue that appears later in the primary sequence. Thus, we capture the positional bias and N-to-C directionality of the fold architecture. The frequency of each amino acid pair was recorded to capture the inherent directionality of the structural logic.

#### 2.2.4. Frequency of Each Pair of Amino Acid in Contact

We calculate the Euclidean distance, with all C_α_-only residues starting from the N terminus and moving to the C terminus. A 20 × 20 matrix is formed by summing all residues in contact in our specific defined class, to obtain a matrix of 400 different types of contact. Thus, each element of this matrix, denoted by **SM_ij_**, can computed as

**SM_ij_** = ∑(**A_i_A_j_**)

where **A_i_A_j_** is the frequency of residue **A_i_** contact with residue **A_j_**. Here, **i** and **j** vary from 1 to 20. Therefore, we started from an initial set of all-α, all-β, α+β, and α/β, with 118, 114, 127, and 126 protein first subunits, respectively (see Figure S1). For each contact in a map (XY) between any two residues **A_i_** and **A_j_**, **Ai** belonged to an X specific structural class, and **Aj** belonged to a Y same structural class element and was always **i** < **j** (**i** and **j** being their respective positions in the primary sequence of the same subunit). To clarify our further definitions, will refer each contact entry in a map with the notation

**Z_1_** [**A_1_** **S_1_**]|**Z_1_** [**A_2_** **S_1_**]

where **A_1_** and **A_2_** are the first (N-nearest) and the second (C-nearest) residue types selected among the 20 possible types of AAs, **S_1_** is the specific structural class which belongs to, and **Z_1_** is the subunit fragment where the contact data came from only for the first subunit.

#### 2.2.5. Generation of Asymmetric Frequency Matrices

Intra-protein contacts were evaluated using all residues with C_α_-only representation. A contact was defined when any residue **i** was within a spatial distance of 3–5 Å from any residue **j**, provided that they satisfied a short-range sequence separation constraint of ∣i − j∣ ≥ 6. For each structural class, a 20 × 20 RRCFM was constructed. Each cell (**A_1_**, **A_2_**) in the matrix represents the frequency of contact between amino acid type **A_1_** and amino acid type **A_2_**. The matrices were maintained as asymmetric entities, where the upper triangle represents N-to-C terminal directionality, and the lower triangle represents C-to-N. This preserves the positional bias of the residues and the topological ‘handedness’ of the tertiary packing (see Supplementary Tables S1–S4). The rows (**i**) represent the positional bias of the amino acid residues appearing earlier in the primary sequence, and the columns (**j**) represent the amino acid appearing later in the sequence. Each cell (**A_1_**, **A_2_**) represents the RRCF between residue type **A_1_** and **A_2_** across the all-α, all-β, α+β, and α/β classes, with 118, 114, 117, and 126 PDB proteins. Residue–residue contact matrices were visualized as two-dimensional heatmaps to evaluate the differences between raw frequencies and standardized values across the structural classes. Because raw contact counts exhibited a highly skewed distribution with maximum values near 2000, a logarithmic transformation (log_10_(Value + 1)) was applied to resolve low-frequency boundaries and high-density cores on a unified scale.

#### 2.2.6. Intra-Class Z-Score Normalization Analysis

By applying Z-score normalization to our RRCF data, we transformed raw counts into a standardized measure of structural deviation, obtaining a NRRCFM. We identify special or statistically significant interactions within a specific architecture; raw frequencies (X) were normalized to Z-scores (Z). Z acts as a filter to separate background noise from the structural anchors that define the fold:

$$Z_{ij}=\frac{X_{ij}-{µ}_{global}}{\sigma_{global}}$$

where μ_global_ and σ_global_ represent the mean and standard deviation of all pair frequencies within that specific class. This step identifies class-specific blueprints, highlighting anchors that are overrepresented (Z > 1.96) regardless of the total amino acid abundance (see Supplementary Tables S5–S8). To ensure robust and length-independent normalization, we established a global structural baseline using a non-redundant dataset of 118, 114, 117, and 126 PDB proteins with pairwise sequence identity. To eliminate oligomeric bias, only the first subunit of each protein was considered. Only carbon-alpha contact matrices were filtered to include long-range interactions satisfying sequence separation and physical distance. A single global mean (μ_global_) and standard deviation (σ_global_) were then computed by pooling all valid contacts across this diverse multi-protein ensemble. Z-score normalization was subsequently applied using these parameters, evaluating contact frequencies against a representative background of native single-chain structures. For the normalized intra-class datasets, a linear divergent scale was implemented, with fixed color limits symmetrically bounded between −1 and 1. This ensured that over-represented (positive up to 1) and under-represented (negative down to −1) interactions were highlighted with equal statistical weight around the zero baseline.

#### 2.2.7. Violin Plot Analysis

To further understand the distribution of RRCF in the structural classes (all-α, all-β, α/β, and α+β) of hydrolase proteins, we have presented our datasets as violin plots, a hybrid between a box plot and a kernel density plot, which shows the peaks in our data. This allows us to observe the distribution of our data, where descriptive statistics and the density of each variable are represented. The violin plots represent the distribution of RRCF across different structural classes of the hydrolase superfamily (see Supplementary Figure S2).

### 2.3. Data Analysis

#### 2.3.1. Inter-Class Z-Score Normalization Correlation and Inter-Class Pearson Correlation Analysis

To evaluate the global alignment between our target matrices and the baseline distributions via Pearson correlation analysis, the 2D contact frequency matrices were flattened into 1D vectors. While flattening in image-processing contexts can disrupt local spatial adjacency, this transformation fully preserves meaningful biological information within our statistical framework. Because the operation utilizes strict bijective mapping, every element in the resulting 1D vector permanently retains its explicit, unique residue-pair coordinate (i, j), keeping the structural and stereochemical fingerprint entirely intact. Since the Pearson correlation coefficient is computed strictly on a point-by-point covariance basis, the spatial indexing layout (2D grid versus 1D continuum) does not alter the mathematical or physical outcome of the analysis. Consequently, this approach treats each distal tertiary interaction as a discrete, independent statistical descriptor, which aligns with the established paradigms for constructing and validating knowledge-based potentials. By evaluating the holistic alignment of these vectorized contact networks, our model successfully captures the global integrity of structurally viable protein cores while screening out misfolded decoy conformations.

Therefore, by evaluating the PC between our inter-class Z-score normalization dataset and inter-class RRCFV, we treat each 20 × 20 matrix as a flattened vector of 400 observations. This allows us to correlate the stability logic of one class directly against another. The stability landscape was mapped by correlating the Z-score normalization vectors between structural classes. We utilized the Pearson correlation coefficient (PCC) to quantify the degree of rule-sharing between architectures. The PCC in protein structural classes typically measures the linear relationship between specific features like amino acid composition, hydrophobicity scales, or solvent accessibility and their fold patterns. In this study, it allows us to correlate the stability logic of one class directly against another. The correlation coefficient is acquired by applying the following formula:

We have two vectors *X* = ($x_{1},x_{2},\ldots ,x_{n}$) and *Y* = ($y_{1},y_{2},\ldots ,y_{n}$) in sample size (*n* ×n=20×20)

$$F(XY)=\frac{\sum_{i=1}^{n}\left(x_{i\;\;}-\bar{x}\;)(y_{i}-\bar{y}\right)}{\sqrt{\sum_{i=1}^{n}{\left(x_{i\;\;}-\bar{x}\;\right)}^{2}{\left(y_{i\;}-\bar{y}\right)}^{2}}}$$

where $x_{i}\;\mathrm{and}\;y_{i}$ are the individual contact frequency indexed with *i*:

$$\bar{x}\;=\;\frac{1}{n}\sum_{i=1}^{n}x_{i\;}\;$$

$$\bar{y}=\frac{1}{n}\sum_{i=1}^{n}y_{i\;}$$

#### 2.3.2. Double-Stage Pearson Correlation Analysis

The mathematical innovation of our study involves a two-stage application of the PCC to our RRCFM and NRRCFM. In Stage 1, we performed an intra-class symmetry analysis (rsym) to quantify internal determinism, in which the upper triangle of a class matrix was correlated against its own lower triangle. High rsym values indicate a high degree of structural symmetry and predictable packing within that class. In Stage 2 we performed an inter-class divergence analysis (rdiv) to identify the universal divergence pattern (UDP), in which the aggregated matrices of different classes were correlated against one another. This stage identifies which residue pairs are universal (high positive r) and which have a negative r.

Supplementary Figures S3–S6 were generated from the data in Supplementary Tables S1–S8, which were obtained by applying Stage 1: the PCC of RRCFM and NRRCFM between the upper triangles (where i comes before j) and the lower triangles (where j comes before i). We obtained eight PCC matrices of structural classes, which each contain 400 values. After excluding the 20 diagonal entries representing self-interactions and isolating the symmetric higher triangle, we retain exactly 190 unique values for each structural class.

Supplementary Figures S7–S12, were obtained by applying Stage 2, which involved calculating the PCC of RRCFM and NRRCFM among the four structural classes and subsequently between the lower and upper triangles using the data from Supplementary Tables S1–S8. Specifically, this entailed pairwise comparisons between matrices, such as Table S1 versus Table S2, and Table S1 versus Table S3, Table S1 versus Table S4, Table S2 versus Table S3, Table S2 versus Table S4, Table S3 versus Table S4, Table S5 versus Table S6, Table S5 versus Table S7, Table S5 versus Table S8, Table S6 versus Table S7, Table S6 versus Table S8, and Table S7 versus Table S8. Consequently, we applied PCC between the lower triangle and upper triangle of all of them, obtaining twelve matrices which each contain 400 values; but because they are symmetric, we retain 190 unique values, as previously described. Therefore, we evaluated PCC in each structural class separately from other structural classes and between each pair of structural classes to measure the linearity of this relationship and determine if there is any similarity in internal structural patterns. The PCC figures, both raw and normalized, show the same results. Given the consistency between the raw and normalized results, and to avoid redundancy, only one representative dataset is displayed in the manuscript. The alternative set of figures, along with extended methodological details, is provided in the Supplementary Materials. For the normalized intra- and inter-class datasets, we applied a linear divergent scale with fixed color limits symmetrically bounded between −1 and 1. This visualization ensures that over-represented (positive up to 1) and under-represented (negative down to −1) interactions carry equal statistical weight around the zero baseline.

#### 2.3.3. Identification of Conserved Statistical Fingerprints in Protein Architecture

A universal Pearson correlation (UPC) analysis was performed to evaluate the linear relationship between the RRCF and the average Z-scores across the four structural classes (all-α, all-β, α+β, and α/β) of the hydrolase superfamily. A cross-class correlation vector was constructed to identify high-coefficient clusters. The common fingerprints were defined as RRCF patterns that maintained high correlation and significant Z-scores across multiple systems, indicating universal structural constraints regardless of specific fold topology. In this subsection we treat each 20 × 20 matrix as a flattened vector of 400 observations, as we treat it in the Section 2.3.1.

#### 2.3.4. Spatial Mapping of Independent Hydrophobic Pockets

To characterize the local packing architecture of the ABHD6 monomeric subunit (Chain A), the selected residue-residue contact network was segmented into two distinct spatial domains or pockets, alongside a central core, based on sequence proximity and structural density. Molecular rendering was performed on a pure white background using customized high-saturation RGB coloring for the target sidechains to maximize contrast. Local solvent-accessible surface areas were generated independently for each subdomain to prevent topological fusion. Pocket 1 (comprising Leu75 and Val101) was rendered using a light orange semi-transparent surface (55% transparency), while Pocket 2 (comprising Tyr257 and Leu176) was rendered in a sky-blue surface. The central core encompassing residues Leu144, Leu168, Leu170, Val171, Ile272, and Ile273 was highlighted using dark blue sticks to visually differentiate it from the peripheral pockets. Backbone traces were maintained as dark gray cartoons, and interacting sidechains were visualized as uniform bright blue sticks, explicitly disabling element-specific heteroatom coloring to ensure visual consistency.

### 2.4. Statistical Analysis

All statistical analyses were performed using RStudio (2025.05.1 and 2026.04.0). The following packages were used: base (4.3.2), compiler (4.3.2), grid (4.3.2), splines (4.3.2), lattice (0.21.9), survival (3.5.7), stringi (1.8.1), ggplot2 (3.5.1), Formula (1.2.5), Hmisc (5.1.1), spam (2.10.0), maps (3.4.1.1), fields (17.3), data.table (1.14.8), dplyr (1.1.4), RcolorBrewer (1.1.3), ellipse (0.5.0), corrplot (0.95), vioplot (0.5.1), Ggally (2.2.1), tidyverse (2.0.0), FactoMineR (2.14), factoextra (1.0.7), openxlsx (4.2.8.1), tidyr (1.3.1), and bio3d (2.4.5). We used the R Base package to generate heatmaps, violin plots, corrplots, and correlograms (Supporting Information). The ggplot2, vioplot, corrplot, and GGally R packages were used to generate most of the figures presented in the manuscript. The *p*-value is symbolically encoded at the levels of 0.05 (*), 0.01 (**), and 0.001 (***) to indicate statistical significance.

## 3. Results

### 3.1. Evaluation of Intra-Class Patterns and Z-Score Intra-Class Patterns in Four Structural Classes of Hydrolase Proteins

In this study, we have evaluated the occurrence patterns of RRCF in non-redundant datasets of the all-α, all-β, α/β, and α+β structural classes of hydrolase proteins. We have represented each of them in an independent map of 20 × 20 RRCFMs. The frequency of neighboring amino acid (AA) composition of each residue with all residues from N-terminus to C-terminus is calculated as described in Section 2. The calculated values for the 475 representative proteins in the structural classes, all-α class (118 PDB), all-β class (114 PDB), α/β class (117 PDB), and α+β class (126 PDB), are given in Supporting Information Tables S1–S4. Each table represents the frequency neighboring of all AAs with all AAs in each structural class. The defined sequence separation was enforced to eliminate the signal from local secondary structure motifs. Thus, by isolating residues that are far apart in sequence but close in 3D space, we are identifying the non-local interactions that dictate global stability. The asymmetric 20 × 20 matrices capture the directionality (N-to-C) of the fold, revealing that protein folding is a vectorial process.

**Figure 2:**
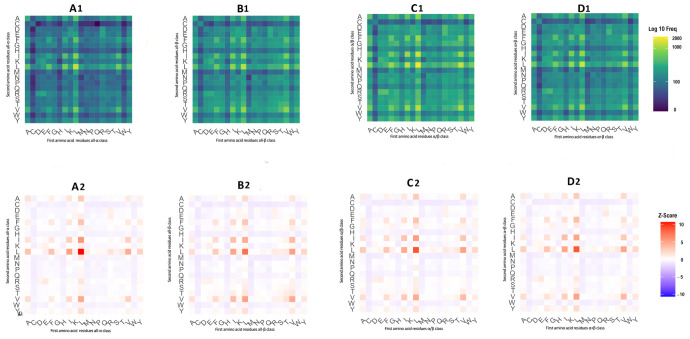
Architectural conservation in diverse hydrolase protein classes: utilizing contact frequency heatmaps to identify stable allosteric hubs for lead discovery. 1: A heatmap shows residue–residue contact frequencies for all the residue types. 2: Normalization Z-score heatmaps for four structural class of hydrolase proteins, from N-terminus to C-terminus. (**A1**,**A2**): all-α class (118 PDB); (**B1**,**B2**): all-β class (114 PDB); (**C1**,**C2**): α/β class (117 PDB); and (**D1**,**D2**): α+β class (126 PDB). Yellow: high contact frequency; dark blue: low contact frequency; red: high Z-score; blue: low Z-score.

Figure 2 provides a clear bridge among protein topology, evolutionary conservation, and structural energetics stability, a critical breakthrough for rational drug discovery. Our results identify a topological skeleton shared across hydrolase proteins, regardless of their fold class. By normalizing physical contact maps with statistical Z-scores (Supporting Information Tables S5–S8), the research defines a statistical representation of structural energetics that distinguishes structural noise from the energetic anchors that are essential for protein function and draggability. The heatmaps (A1–D1) reveal a highly specific hierarchy of amino acid interactions, where the hydrophobic residues emerge as the primary structural drivers. The strong affinities are dominated by hydrophobic–hydrophobic clusters and charge–charge interactions. While all classes (all-α, all-β, α/β, α+β) share a similar top-tier contact pattern, class all-β shows a significant increase in overall RRCFM compared to all-α. Mixed classes (C1 and D1) maintain these core patterns but exhibit specific intensity shifts, suggesting that, while the skeleton is universal, the density is class-specific. Our results translate 2D heatmaps into a 3D stability profile, which identifies statistical representation of structural energetics. Dense, high-frequency regions in the RRCFM indicate structurally optimized, low-energy contact clusters. Within a knowledge-based potential framework, these clusters represent the primary statistical energy holding the backbone scaffold together. Furthermore, the strong congruence between high raw contact frequencies and elevated Z-scores indicates a highly restricted conformational entropy. These interactions do not represent stochastic or random encounters; rather, they map rigid evolutionary and geometric constraints that limit local backbone flexibility to ensure overall fold stability [57]. Consequently, this highly cooperative, low-entropy structural scaffold creates a well-defined topological fingerprint. In drug discovery, such geometric rigidity signals a highly stable target site, where binding pockets are structurally anchored and less likely to undergo unpredictable conformational shifts. Ultimately, the high overlap between the RRCFMs (A1–D1) and the class-specific Z-score matrices (A2–D2) serves as a robust mathematical filter to identify these critical structural hotspots.

The Z-score successfully distinguishes between incidental spatial neighbors and evolutionarily constrained structural hubs. Overlapping regions characterized by both high raw contact frequencies and elevated Z-scores are identified as the primary energetic anchors contributing to overall fold stability. Because these overlapping nodes function as critical topological bottlenecks, disrupting a high-Z-score residue is statistically more likely to compromise the structural integrity of the entire enzyme network. Consequently, these sites represent premier candidates for allosteric drug design, offering a direct mechanism to map and target distal communication pathways. Therefore, the strong congruence between the physical topography of the RRCFM and the statistical constraints of the class-specific Z-score distributions confirms that these short-range (C_α_) interactions constitute the fundamental building blocks of protein architecture. Ultimately, this framework provides a robust topological roadmap of structural stability, precisely identifying which enzymatic sites are the most viable targets for therapeutic intervention.

Furthermore, the RRCFMs and NRRCFMs present both symmetrical and asymmetrical topologies when comparing their respective upper and lower triangles (A1–D1 and A2–D2). In a C_α_ tertiary network, the statistical evidence that residue i (upstream in the sequence) registers a distinct contact frequency with residue j (downstream) compared to its transpose (X_ij_ ≠ X_ji_) provides a robust topological signal of folding directionality. Conversely, the symmetrical patterns observed across the diagonal isolate reciprocal stability hubs, where specific residue pairs contribute to mutual structural stabilization. The persistence of these dual topological features leads us to conclude that each structural class possesses a defining, invariant topological skeleton. This scaffolding is not merely a trivial byproduct of primary amino acid sequencing, but represents a functional requirement that dictates three-dimensional structural integrity. Consequently, rather than assuming that distinct structural folds operate under isolated geometric constraints, these findings demonstrate that diverse architectures converge upon universal physical packing principles. Our class-specific Z-score normalization successfully unifies these shared constraints under a single, comparative statistical scale.

### 3.2. Class-Specific Stability Profiles

**Figure 3:**
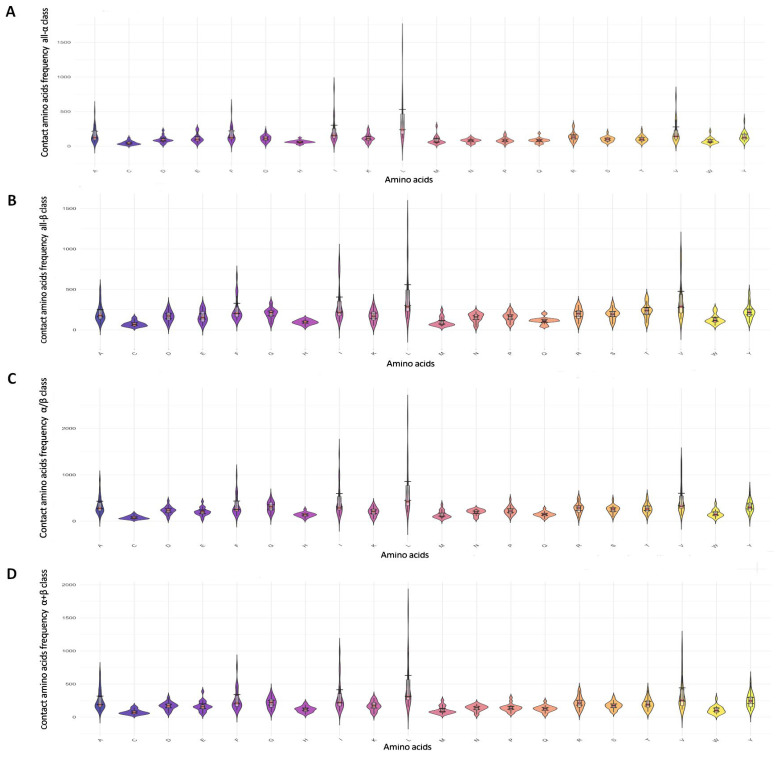
A universal stability score for hydrolase superfamilies: quantifying structural robustness across diverse fold topologies via Residue–Residue Contact Networks. Each class represents a different folding architecture; the *X*-axis represents different types of residue interactions, and the contact frequency on the *Y*-axis is a proxy for how tightly packed the protein is. Violin plots show the distribution of contact frequencies of each AA, with all AAs contained in each structural class of hydrolase proteins from N-terminus to C-terminus. (**A**): all-α class (118 PDB); (**B**): all-β class (114 PDB); (**C**): α/β class (117 PDB), and (**D**): α+β class (126 PDB). The interiors of the violin plots display medians and interquartile ranges, as in a standard box plot, with whiskers indicating the minimums and maximums, along with additional error bars. Each color represents the contact frequency of a specific AA with all other AAs.

The variance of contact frequencies was mapped for each of the four structural classes of hydrolase proteins (Figure 3A–D). We create a violin plot for the total distribution of all amino acid pairings. By comparing the difference in contact density (Y-axis), we found that the violin height reflects evolutionary preference. If Leu is higher on the *Y*-axis, it is because nature uses it more frequently to stabilize the core. Panels C (α/β class) and D (α+β class) show significantly higher maximum contact frequencies (reaching up to 2000) compared to panels A (all-α class) and B (all-β class), which peak around 1000–1500. α/β class proteins are generally more densely packed and exhibit more complex internal contact networks. Hydrolase enzymes are among the most common and successful drug targets, because their dense packing creates stable, well-defined binding pockets. The extremely long tails in panels A, B, C, and D indicate specific residues that act as structural hubs, with an exceptionally high number of contacts. These hubs are critical for the protein’s folding stability. In drug discovery, these are prime sites for allosteric inhibition. If a drug binds near a hub, it can destabilize the entire fold or prevent the conformational changes necessary for function. The stability in this class relies on super-hubs, represented by the violet- and pink-colored spikes that are likely Isoleucine (I) or Leucine (L). In class D, these residues are not just in a core; they are acting as staples that hold two different structural worlds together. These proteins showing high frequency have a very high unfolding barrier. Because the residues are so tightly packed, it takes a massive amount of thermal energy to break those contacts.

From Figure 3 we can deduce the presence of the UG that comes from the striking similarity in the marginal distributions (the overall shape) of the four violins. We notice that for the four violins of the four classes (A, B, C, and D), the widest part of the violin, the bulge, occupies roughly the same vertical range on the *Y*-axis. This might indicate that, regardless of the structural architecture (whether it is mostly helices or sheets), there is a fixed physical preference for how often residues touch within that 3–5 Å window. Furthermore, the UG suggests that nature has a goldilocks zone for packing density. If a synthetic drug’s contact frequency falls outside this common bulge, it is likely biologically unnatural and prone to immediate degradation. In addition, all four classes exhibit vertical tails or spikes that taper off as they move toward higher contact frequencies. While classes C and D might have slightly longer spikes, the decay rate (how the frequency drops off) is mathematically similar across the board. This tells us that there are structural anchors that follow a universal distribution of strength. There are many weak interactions and a very specific, limited number of high-frequency master anchors across all structural classes of the hydrolase enzyme family.

Finally, by looking closely at the center of each violin, the internal markings (representing the middle 50% of the data) align horizontally across A, B, C, and D. This overlap of the interquartile range (inner box) is the statistical proof of UG. This means that the expected stability of a protein is not a random variable; it is governed by a shared physical core. For example, from a drug-design perspective, if class A and class D share this core grammar, we can use the same mathematical stability constants to predict the shelf life of an enzyme-class drug.

By seeing this overlap, we realize that instability is not usually caused by a protein having different grammar, but by a protein failing to meet the universal standard. Therefore, industrial pharmaceutics might use this UG as a control chart, where they can use the overlapped area of these four violins as the standard for stability. For instance, if we design a new enzyme, and its violin plot shows a bulge much lower or narrower than these four, we have identified a stability gap. The overlap in our figures proves that tertiary determinism (the ‘why’ of the fold) is not class-dependent, but physics-dependent. This allows us to create a USG that can be applied to any enzyme superfamily, regardless of its specific 3D shape.

Supplementary Figure S2 shows the statistical distribution of contact frequencies across the 20 standard amino acids, resolved by structural class (all-α, all-β, α/β, and α+β). For each class, the median and interquartile range (IQR) are explicitly contrasted. As observed, hydrophobic residues such as Leucine (L) and Valine (V) exhibit the highest median contact frequencies and widest IQRs, signifying their dynamic role in space-packing constraints. Conversely, the α/β class systematically displays elevated medians and broader IQR profiles compared to the all-α and all-β classes across most residues (e.g., G, I, R, Y). This precise quantification maps the baseline variance and overlap statistics required to decode the universal structural grammar of each residue type.

### 3.3. Evaluation of the Inter-Class Z-Score Correlation and Inter-Class Pearson Correlation

**Figure 4:**
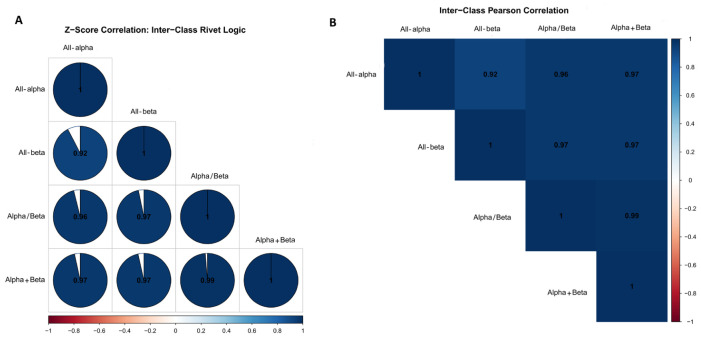
(**A**): Inter-class stability correlogram. (**B**): Inter-class PC vectors. (**A**): The plot represents the PC of NRRCFV among the four structural classes. (**B**): The stability landscape was mapped by correlating the Z-score vectors among the four structural classes.

To compare the global stability logic between architectures, each 20 × 20 Z-score matrix was flattened into a directional vector of 400 elements. The PCC was then calculated between these vectors for each pair of structural classes. By correlating the entire vector, we captured the global stability grammar. Thus, we bridge the gap between basic protein geometry and the systematic logic of protein folding. We have moved beyond simple proximity to address the vectorial nature of how residues pack together. The 400 pairs of the all-α, all-β, α/β, and α+β classes were treated as a single vector to assess the inter-class Z-score correlation and inter-class PCC of RRCF. Thus, we found that the signal (the high-Z anchors) and r = 0.99 correlation prove that the main scaffolding shared the same biological law. These qualitative rules are the same as ‘universal grammar’. In statistical terms, we found that the patterns are correlated. By performing this analysis using a contact frequency matrix, we are essentially comparing how frequently specific amino acid pairs interact across different structural classes. A high PCC between two classes suggests they share structural grammar, meaning the energetic and geometric rules governing their folding are similar, which offers significant structural insights that can be leveraged in fragment-based drug design (Figure 4A,B). Our Z-score correlation plot provides significant insight into protein physics by demonstrating extremely high correlations across all four classes. This proves that, although the raw frequencies may differ, the statistical logic underlying which amino acids act as anchors is almost universally shared across all folds. Our results reveal near-perfect inter-class correlations ranging from 0.99 to 0.92, proving that distal packing rules are a conserved physical constant across the proteome. These PCC values indicate that, if a specific amino acid pair acts as a structural anchor in all-α proteins, it is highly likely to act as a structural anchor in all-β, α/β, and α+β proteins as well, helping us to identify universal stability grammar.

This framework provides a mathematical foundation for mapping the stability landscape, allowing for distinction between this universal grammar and the subtle, fold-exclusive motifs that are critical for achieving high drug specificity.

Additionally, the correlation between α/β and α+β is nearly perfect (r = 0.99). This suggests that, at the level of distal physical interactions (∣i − j∣ ≥ 6), these two classes are mathematically identical in their selection of stabilizing residues, which reveals that distal structural anchors are governed by a conserved physical logic that transcends secondary structure arrangement. Therefore, the high correlation in contact frequencies among the four structural classes suggests that the hydrophobic core packing and salt-bridge patterns may be identical. A drug fragment that stabilizes a specific contact in one class is likely to be effective in the others. In the context of drug design, a high r-value confirms the statistical robustness of the chemical similarity between two protein classes, making it a reliable basis for scaffold hopping.

### 3.4. Distribution of Internal Structural Determinism

**Figure 5:**
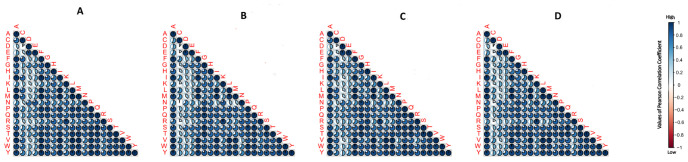
Universal symmetric and asymmetric patterns of tertiary contact networks. Correlograms of inter-class contact frequencies. The plots display the Pearson correlation coefficient matrices calculated for each pair of structural classes in hydrolase proteins. Panels illustrate both raw contact frequencies and their corresponding Z-score normalized counterparts for all amino acid types, from N-terminus to C-terminus. (**A**): All-α class (118 PDB), (**B**): all-β class (114 PDB), (**C**): α/β class (117 PDB), and (**D**): α+β class (126 PDB). Colors indicate different values of the correlation coefficient according to the scale bar reported. Blue is used to indicate positive correlation, while red is used to indicate negative correlation. The lighter the tone used, the less significant the corresponding correlation. The filled fraction of the circle in each of the pie charts corresponds to the absolute value of the associated PCC.

To better explore the relationship between the RRCFs of each AA with all AAs in all structural classes of hydrolase proteins from N-terminus to C-terminus, we applied Pearson’s correlation method to our dataset, both raw and normalized. The map of RRCFMs and Z-scores of RRCFMs for each structural class of hydrolase proteins was evaluated as described in Section 2. Figure 5A–D and Supplementary Figures S3–S6 show that the values of PCC are principally represented by a positive linear correlation with respect to several RRCFs and Z-scores of RRCFMs in the four structural classes of hydrolase proteins.

Obviously, we see a very strong positive correlation coefficient corresponding generally to hydrophobic RRCFs such as Ala, Phe, Gly, Ile, Leu, Met, Pro, Val, and Trp with polar residues Gln, Ser, Thr, and Tyr in all structural classes. And we noticed slight differences when we compared the PCCs of RRCFs of all structural classes of hydrolase proteins. They are mostly represented by a very strong to strong positive linear correlation, rather than a moderate to weak positive linear correlation. Therefore, all-α class, all-β class, α/β class, and α+β class hydrolase proteins have very strong positive correlation coefficients of 48.5%, 45.5%, 50%, and 46.5%, respectively. We found that nearly half of our dataset follows a rigid, symmetric ‘rule’. These are likely the highly conserved, evolutionary optimized tertiary contacts that define the fold. For drug discovery, these are high-confidence targets. Furthermore, they have strong positive correlation coefficients of 36%, 34.5%, 31.5%, and 30%, respectively. These represent the stable architecture that still allows for some natural breathing or conformational change. In addition, we show moderate positive correlation coefficients of 11%, 14%, 14.5%, and 18.5%, respectively, where the asymmetry of RRCF is becoming apparent. Finally, weak to very weak positive correlation coefficients of 4.5%, 6%, 4%, and 5%, respectively, were observed. This small fraction represents high asymmetry. These are high-risk targets, because their tertiary network is unpredictable.

Therefore, those outcomes reveal for the first time the presence of RRCF fingerprints in structural classes of hydrolase proteins. By comparing the upper and lower triangles of the class-specific matrices (reflecting the symmetry of N-to-C vs. C-to-N tertiary contacts), a four-tier stability distribution emerged, uncovering the hierarchy of determinism. We have created physical grammar for proteins, and our r-values dictate how amino acids form a stable, functional machine. Instead of viewing each class as a single average correlation, this analysis uncovers a spectrum of stability that reflects the fractal nature of protein stability. This distribution represents the internal diversity of the all-α class (118 PDB), all-β class (114 PDB), α/β class (117 PDB), and α+β class (126 PDB) PDB structures within each class. The similarity in these percentages across the four structural classes suggests a universal law of protein packing. It implies that nature constructs all folds (whether helical or sheet-based) using a similar ratio of rigid anchors vs. flexible connections. While the geometry of the contacts differs between α and β, the statistical distribution of their stability is a fundamental property of protein-based matter, which reveals a universal determinism.

In addition, our analysis reveals a universal distribution of structural determinism across all protein classes. Approximately 85% of all tertiary networks (strong to very strong) operate under a symmetric rigid-rule regime. However, the remaining 23% (low to moderate) represent a plasticity reservoir correspond to some contact with Cysteine, Aspartic Acid, Glutamic Acid, Asparagine, Arginine, and Lysine. The fact that this distribution is conserved across α and β classes suggests that the ratio of rigidity to plasticity is a fundamental constraint of protein architecture, regardless of the specific secondary structure components. Therefore, this similarity would be helpful for predicting drug stability. It means that the rules for stability are robust and transferable.

### 3.5. Inter-Class Divergence of the All-α Protein Class with Respect to the All-β, α/β, and α+β Proteins Classes Using Pearson Correlation

**Figure 6:**
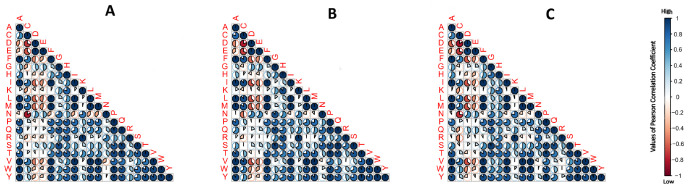
Universal divergence patterns of tertiary contact networks. Correlograms of inter-class contact frequencies. The plots display the Pearson correlation coefficient matrices calculated for each pair of structural classes of hydrolase proteins. Panels illustrate both raw contact frequencies and their corresponding Z-score normalized counterparts for all amino acid types between two structural classes of hydrolase proteins. (**A**): All-α (118 PDB) vs. all-β (114 PDB), (**B**): all-α (118 PDB)/α vs. β (117 PDB), and (**C**): all-α (118 PDB) vs. α+ β class (126 PDB). Colors indicate different values of the correlation coefficient according to the scale bar. Blue is used to indicate positive correlation, while red is used to indicate negative correlation. The lighter the tone used, the less significant the corresponding correlation. The filled fraction of the circle in each of the pie charts corresponds to the absolute value of the associated PCC.

To delve further into internal geometry and gain a deeper understanding of the all-α, all-β, α/β, and α+β structural classes of hydrolase protein, we evaluated the relationship between RRCF and Z-score normalization of RRCF for each AA in the all-α protein class with regard to all AAs in the all-β, α/β, and α+β proteins classes of the hydrolase superfamily from the N-terminus to the C-terminus. We applied the PCC between them as described in the Materials and Methods (Figure 6A–C and Supplementary Figures S7–S9).

The results obtained by cross-correlating the aggregated matrices and applying Stages 1 and 2 of PCC between structural classes revealed a UDP. We found that the values of PCC of RRCF and Z-score of RRCF for the all-α protein class with respect to several RRCFs and Z-score of RRCFs for the all-β, α/β, and α+β structural classes of hydrolase proteins are represented by negative and positive linear correlations. We can perfectly differentiate a very strong to strong positive linear correlation corresponding principally to hydrophobic AAs with regard to polar, hydrophobic, and basic AAs.

Therefore, with regards to the all-β, α/β, and α+β classes of hydrolase proteins, the all-α class presents very strong to strong positive PCCs of 46%, 46%, and 44%, respectively. In addition, there was a moderate positive PCC of 16% with regard to the three classes. Further, there were weak to very weak positive PCCs of 16%, 19%, and 16%, respectively, and negative PCCs of 22%, 19%, and 24%, respectively. Therefore, the statistical profile remained approximately similar across all inter-class comparisons. This indicates that the mathematical distance between a helix-dominant fold and a sheet-dominant fold is constant. The divergence is not random; rather, it follows a conserved redistribution of residue contacts, where the inter-class divergence identifies universal vs. fold-specific stability fingerprints. We found a spectrum of stability which can be considered to indicate the fractal nature of protein stability. Our results reveal a preserved structural pattern related to RRCFs and Z-score of RRCFs of the all-α class with regard to the all-β, α/β, and α+β classes of hydrolase proteins, which are represented principally by very strong and strong positive linear correlation values. Those preserved fingerprints reflect the internal geometry of each structural class, which helps us to understand how similar the RRCFs and Z-score of RRCFs of each structural class are with regard to the other structural classes.

### 3.6. Inter-Class Divergence of All-β, α/β, and α+β Protein Classes Using Pearson Correlation

**Figure 7:**
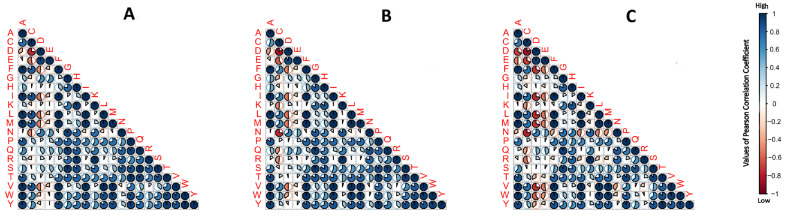
Universal divergence patterns of tertiary contact networks. Correlograms of inter-class contact frequencies. The plots display the Pearson correlation coefficient matrices calculated for each pair of structural classes of hydrolase proteins. Panels illustrate both raw contact frequencies and their corresponding Z-score normalized counterparts for all amino acid types between the compared classes. (**A**): All-β (114 PDB) vs. α/β (117 PDB), (**B**): all-β (114 PDB) vs. α+β (126 PDB), and (**C**): α/β (117 PDB) vs. α+β class (126 PDB). Colors indicate different values of the correlation coefficient according to the scale bar. Blue is used to indicate positive correlation, while red is used to indicate negative correlation. The lighter the tone used, the less significant the corresponding correlation. The filled fraction of the circle in each of the pie charts corresponds to the absolute value of the associated PCC.

To deepen comprehension of the internal geometry of the all-β, α/β, and α+β structural classes of the hydrolase superfamily from N-terminus to C-terminus, we also assessed the correlation between the RRCFs and Z-scores of each AA in the all-β protein class with regard to all AAs in the α/β and α+β classes, as well as the α/β with regard to α+β classes of hydrolase proteins. We implemented the PCC between them as described in Section 2. Figure 7A–C and Supplementary Figures S10–S12 show that the PCC values for RRCFs and their corresponding Z-scores also exhibit negative and positive linear correlations. Our outcomes also disclose the existence of a structural correlation between the structural classes; especially, the higher correlations uncover the hidden structural patterns common in those classes, as seen in the previous subsection. We can perfectly differentiate a very strong to strong positive linear correlation corresponding principally to hydrophobic AAs with regard to polar, hydrophobic, and basic AAs. Therefore, the percentages of contact frequencies exhibiting very strong to strong positive PCCs for the all-β class with regard to the α/β and α+β classes, and for the α/β class with regard to the α+β class of hydrolase proteins, are 50%, 47%, and 43%, respectively. In addition, they present moderate positive correlation coefficients of 12%, 13%, and 9%, respectively. Further, we show weak to very weak positive PCCs of 24%, 20%, and 21%, respectively and negative PCCs of 15%, 20%, and 27%, respectively. Therefore, the statistical profile remained approximately similar across all inter-class comparisons. This indicates that the mathematical distance between a helix-dominant fold and a sheet-dominant fold is constant. The divergence is not random; rather, it follows a conserved redistribution of residue contacts. Furthermore, we also uncovered a spectrum of stability which can be considered to indicate an approximate fractal nature of protein stability in inter-class stability and identifies universal vs. fold-specific stability fingerprints.

### 3.7. Identification of Conserved Statistical Fingerprints in Hydrolase Architecture

To better understand the protein structure, it is necessary to improve our comprehension of the RRCFs of the four structural classes of hydrolase proteins. In Section 3.3, the highest correlation was obtained, ranging from 0.99 to 0.92. In this section, we shift from a macro-level analysis to a micro-level analysis to reveal hidden structural patterns common to these four structural classes of the hydrolase superfamily.

**Figure 8:**
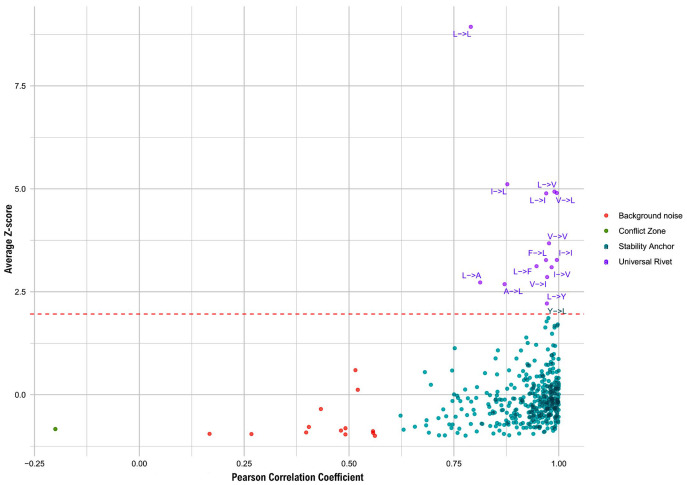
Conserved statistical fingerprints in proteins architecture. Scatter plot decoding the USG of long-range interaction networks across four structural classes of hydrolase proteins. The *X*-axis represents the UPC of RRCFs, quantifying the degree of conservation across the four structural classes, and the *Y*-axis represents the average Z-score of RRCFs, indicating their statistical deviation from random interaction models. A horizontal red dotted line is positioned at Z = 1.96, establishing the threshold for statistical significance (*p* < 0.05). Points plotted above this boundary represent residue interactions that occur significantly more frequently than expected by chance. Each color represents a specific cluster.

The results obtained are presented in Figure 8. Based on the distribution of the violet points in this figure, the data show that thirteen RRCFs are common to all four structural classes of hydrolase proteins, representing 3.25% of contacts dominated by hydrophobic–hydrophobic pairs (Leu-Leu, Ile-Leu, Leu-Val, Leu-Ile, Val-Leu, Val-Val, Phe-Leu, Ile-Ile, Leu-Phe, Ile-Val, Leu-Ala, Val-Ile, and Leu-Tyr). Those with a higher average Z-score (Z > 1.96) and UPC values ranging from very strong to strong positive correlation are considered universal rivets or universal druggable pockets. Furthermore, the distribution of the blue points shows that 373 RRCFs are common to all four structural classes, with an average Z-score of less than 1.96 and UPC values ranging from very strong to strong positive correlation, representing 93.5% of RRCFs. We consider these to be stability anchors. The distribution of the red points shows that 12 RRCFs are common to the four structural classes, with an average Z-score of less than 1.96 and UPC values ranging from moderate to very weak positive correlation, representing 3% of RRCFs. We consider these to be background noise. Finally, the green points are positioned in all four structural classes, have an average Z-score of less than 1.96, and have lower UPC values, indicating a very weak negative correlation, representing 0.25% of RRCFs. We consider this to be the conflict zone.

Therefore, as can be seen from Figure 8, the four structural classes of hydrolase proteins conserve their architectural fingerprints, sharing 97% of identical residue-residue contacts in their spatial architecture. This exceptionally high overlap demonstrates that they govern their folds through a deeply unified structural logic. For instance, if a particular amino acid pair plays a key role in all-α proteins, it typically plays an equally significant role in the other protein classes, highlighting the high degree of structural similarity. Although the four structural classes of hydrolase proteins are categorized differently, the figure shows that they share a very similar underlying structural foundation, which is considered USG.

### 3.8. Topological and Spatial Characterization of ABHD6 Core Contacts

To identify the fundamental architectural constraints governing the tertiary structure of the ABHD6 monomer (Chain A), we evaluated the residue–residue contact frequencies (RRCFs) filtered by strict spatial and statistical criteria. Applying a sequence separation window of ∆i ≥ 6 minimized trivial local secondary structure constraints, while an exclusive 3D Euclidean distance window of 3–5 Å isolated closely packed atom pairs within the protein core. To isolate true structural signals from baseline amino acid abundance, intra-class Z-score normalization was applied. Out of the total contact network, a highly over-represented subset of five core interactions was identified as statistically significant, exceeding the critical threshold of Z > 1.96 (*p* < 0.05).

**Figure 9:**
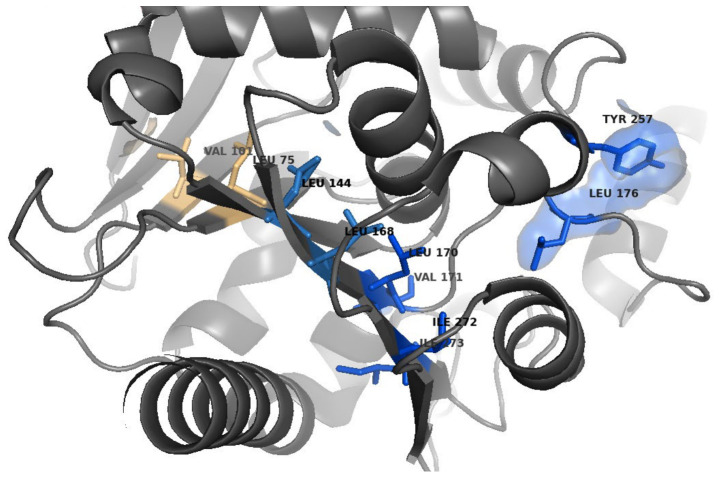
Segmented local pocket representation of key hydrophobic core contacts in ABHD6. The tertiary structure of the functional monomer (Chain A) is shown in dark gray cartoon format. Target residue–residue interactions are mapped as uniform bright blue sticks enclosed within two distinct, independent, semi-transparent molecular surfaces (55% transparency), while the central core is highlighted with dark blue sticks. The light orange and sky-blue surfaces define localized micro-pockets (specifically isolating the Tyr257 and Leu176 interaction on the right) or packing domains that isolate specific long-range and short-range structural correlations. Inter-atomic Euclidean distances between Cα positions are indicated by black dashed lines, with primary residues labeled for spatial orientation. This discrete multi-pocket arrangement illustrates the compartmentalized stabilizing forces operating within the protein core with Z > 1.96.

Spatial mapping of these significant contacts revealed a discrete, compartmentalized arrangement within the protein core, rather than a single, homogeneous packing domain. As shown in Figure 9, these interactions are organized into two independent micro-pockets alongside a central packing hub:

Pocket 1 (Peripheral Stabilizing Hub): Driven by the long-range interaction between Leu75 and Val101 (d = 4.83 Å, ∆_i_ = 26), which anchors peripheral loop regions to the core scaffold.

Pocket 2 (Intermediate Packing Domain): Formed by the tight pairing of Tyr257 and Leu176 (d = 4.89 Å, ∆_i_ = 24), reinforcing the structural integrity of intermediate alpha-helices.

Central Core Domain (Catalytic & Packing Hub): Constitutes a highly dense packing cluster encompassing residues Leu144, Leu168, Leu170, Val171, Ile272, and Ile273. Notably, this central hub exhibits tight packing distances ranging from 4.62Å (Val171–Ile273) to 4.88 Å (Leu170–Ile272), despite vast sequence separations (∆_i_ = 102).

The presence of Ile272 and Ile273 within this central cluster is structurally critical, as these residues are positioned immediately adjacent to the catalytic triad (specifically neighboring Asp278). The tight distance metrics (4.6–4.8 Å) observed across both pockets and the central core correspond to optimal van der Waals contact shells for aliphatic and aromatic sidechains.

## 4. Discussion

In recent decades, maintaining the structure of protein and peptide drugs has become one of the most important goals for scientists. This is because these pharmaceutical proteins are subject to significant physical and chemical instability, necessitating advanced, multi-level stabilization strategies [58,59,60]. In structural biology, the tertiary architecture of proteins is widely accepted to be a structured, deterministic hierarchy, rather than a random cluster of interactions, dictated by the linear amino acid sequence [61,62,63]. This implies that the 3D shapes of proteins can be predicted and modelled with high precision for drug discovery purposes [64,65].

In this study we address a critical bottleneck in structural biology by moving from structural prediction to physical understanding. Figure 1 illustrates our mathematical framework, which demonstrates for the first time that protein tertiary architecture is not a continuum of random interactions, but rather a structured, deterministic hierarchy. The folding process is governed by specific stereochemical rules and thermodynamic principles, resulting in a distinct structure. To capture the long-range packing determinism of the protein interior, a specific distance shell of 3.0 Å < d < 5.0 Å was utilized. This range specifically excludes short-range hydrogen bonding, focusing instead on the non-directional van der Waals forces and hydrophobic desolvation effects that govern the structural logic of the fold. By setting ∣i − j∣ ≥ 6, we exclude contacts within a six-residue window. The frequency matrices capture the tertiary anchors that hold the 3D shape together of the hydrolase enzyme superfamily together. The high-frequency contacts identified in our analysis, specifically Leu-Leu, Ile-Leu, Val-Val, and Phe-Leu, are in full agreement with established structural biology paradigms. These aliphatic and aromatic–aliphatic pairs represent the primary constituents of the hydrophobic core, which constitutes the most evolutionarily conserved region of a protein’s fold. Our findings are externally validated by classical knowledge-based potentials, most notably the Miyazawa–Jernigan (MJ) matrix [24], where these specific pairs are assigned the most favorable interaction energies. Furthermore, the dominance of these clusters aligns with the hydrophobic collapse model [66], which highlights core packing as a primary driver of structural stability. By capturing these specific interactions within the strict, open spatial interval using a coarse-grained (C_α_) framework, our model effectively isolates the fundamental structural scaffolding. These specific residue hubs show significantly lower substitution rates in evolutionary studies [67], confirming that our contact frequency matrix successfully maps onto the functionally critical and structurally conserved core of the protein architecture. However, it is worth noting that a previous study focused on amino acid propensities, identifying class-specific residues, but failed to capture the spatial networks required for ligand stabilization [68].

A key distinction between our scoring approach and comprehensive database resources like the PCA lies in the operational objectives and network abstraction. While the PCA operates as a multi-scale analytical platform that maps every individual atom–atom collision [69], our framework condenses these macro-topological principles into a standardized, coarse-grained residue–residue contact frequency matrix tailored for structural validation and decoy ranking. By defining a valid residue pair encounter exclusively through a sharp carbon-alpha (C_α_) spatial window of 3.0 to 5.0 Å, our model respects the physical principles of excluded volume [70,71]. Because each C_α_ node occupies a fixed physical space along the polypeptide chain, there is an absolute stereochemical upper bound to how many residues can pack into a single local environment. This geometric saturation acts as a natural stabilizer for our data, preventing raw residue–residue contact frequencies from exploding into infinite noise or generating numerical instability across proteins of varying sizes. Crucially, this stabilizing effect provides the robust mathematical foundation required to extract clean statistical potentials. This approach directly expands upon the methodologies established previously in the assessment of amino acid neighborhood preferences using broader residue-based spherical coordinates [72].

In addition, our framework captures a statistical representation of structural energetics grounded in the inverse Boltzmann distribution principle. According to knowledge-based potential paradigms [24], the observed contact frequencies of residue pairs within natively folded structures are inversely proportional to their effective interaction energies. By restricting our coarse-grained network to a strict, open spatial interval between 3.0 Å and 5.0 Å (exclusive of the boundaries), our model focuses on the structural anchors and backbone coordinates that sustain stable core environments. The high-frequency contact pairs identified by our matrix align with the most energetically favorable residues established in traditional statistical potentials, where hydrophobic packing dominates the structural interior [73]. The robust behavior of these frequencies, including their invariance under class-specific normalization and their sensitivity to directionality, demonstrates that these topological networks are not stochastically driven, but instead map the statistical energy landscape that dictates stable protein scaffolding.

By applying PC to contact frequencies, we move beyond 1D composition to 3D interaction patterns, identifying conserved pharmacophoric anchors that simple residue counting overlooks. The PCCs of RRCFM and NRRCFM show symmetrical patterns principally presented by higher RRCF and NRRCF and asymmetrical patterns presented by lower RRCF and NRRCF. When the upper and lower triangles of the contact matrices are compared, it is revealed that protein folding is not a random collapse, but a directional architectural process. The asymmetry and symmetry in the four structural classes suggest that, while helical and beta-sheet segments are internally stable, their global assembly orchestrates stochastic and rigid conformational states, enabling the protein to function. Previous research has shown that the folding process often occurs in a specific direction, typically from the N-terminus to the C-terminus. This is important for achieving the correct functional protein structure [43]. Figure 2(A1)–(D1),(A2)–(D2) and Supporting Information Tables S1–S8 show that the high affinities are dominated by hydrophobic, charged, and polar interactions that correspond to conserved residues and experimental hotspots. These hotspots overlap residues that vibrate with high frequencies at intermolecular binding interfaces. Hydrophobic interactions are widely accepted as the main driving force behind the folding, assembly, and binding stability of biomolecules [74]. Furthermore, polar and charged interactions play a key role in forming binding sites in biological molecules. These interactions often determine the exact location at which molecules such as ligands bind [50,51,52]. Previous studies have validate the proposition that binding and folding are similar processes [75,76]. Therefore, our results provide data that substantiate the earlier proposition that protein binding and protein folding have similar underlying principles.

From a drug discovery perspective, the symmetrical hubs provide the most stable anchor points for ligand binding. Meanwhile, the asymmetrical patterns offer class-defined pockets that can be exploited for selective target inhibition. The interplay between symmetrical structural hubs and asymmetrical functional deviations confirms that the topological skeleton is the primary determinant of hydrolase stability. This provides a clear roadmap for identifying druggable domains within the universal stability score framework as fragment-based drug discovery [57]. Previous research shows that residues involved in high-frequency, non-normalized interactions tend to be conserved and act as folding nuclei. These residues are found in the protein core and, in the case of protein–protein interfaces, establish binding affinity [77].

The total distribution of all amino acid pairings of the four structural classes of hydrolase proteins is represented by violin plots, where the interactions represent the fundamental organizational principles of the proteome. Figure 3A–D show that the overall shape of the plots of the four violins reveals USG. These plots determine a fixed physical preference for how often residues touch within that 3–5 Å window. In the context of predicting the stability, particularly of protein-based drugs, these violin plots provide a wealth of information regarding structural integrity.

Moreover, we shift our focus from general structural similarities to strict NRRCF, in which PCC becomes a measure of evolutionary and energetic conservation. By treating our dataset of 475 pairs as a single vector of all-α, all-β, α/β, and α+β classes and evaluating the inter-class Z-score correlation and inter-class PCC of RRCF, we find the same qualitative patterns. Quantifying the SG shared between folds using PCC yielded a value of 0.99, indicating high correlation between the α/β and α+β classes (Figure 4A,B).

These contact patterns are present in sufficient quantities to allow scaffold hopping, a process that has previously been shown to enhance drug design [78,79]. The inter-class PCC of RRCFV and Z-score normalization of RRCFV yields a similar pattern across diverse pairings. This implies that the transition between an all-α and an all-β fold is governed by a fixed redistribution logic (Figure 4A,B).

In addition, we implemented a double-stage PC framework where we systematically correlated the upper (i < j) and lower (i > j) triangles of our contact frequency matrix. Mathematically, a near-perfect Pearson correlation (r ≈ 1.0) between the two triangles would indicate a symmetric process where asymmetry is merely an indexing consequence. However, our observed divergence in the Pearson correlation, ranging from very strong to very weak across different residue pairs, confirms that the probability of a residue (i) interacting with a downstream partner is not equivalent to its interaction with an upstream neighbor (Xij ≠ Xji). This statistical non-equivalence demonstrates that the connectivity network is highly sensitive to the N-to-C terminal vector. Physically, this asymmetry captures the structural anisotropy of the folding process, which is inherently linked to the temporal sequence of secondary structure formation during ribosome translation and cooperative collapse. Consequently, by maintaining the raw, asymmetric counts, our model successfully preserves the intrinsic vector of the polypeptide chain.

Therefore, we found that, in Stage 1, the PCCs of RRCFM and NRRCFM are asymmetric and symmetric matrices of the all-α, all-β, α/β, and α+β classes. Thus, our results reveal that the quantitative weight or importance assigned to these rules differs slightly between the classes (Figure 5A–D). The fact that ~80% of the intra-class spectrum maintains a very strong to strong correlation or symmetry across all classes, with a significant *p*-value of ≤ 0.001, suggests that the stability rules are highly consistent. Supplementary Figures S3–S6 show that this positional logic is a stable, deterministic feature of the protein fold, revealing the USG. The high correlation and significant *p*-values ≤ 0.001 indicate that specific amino acid pairings occur with the same relative frequency across different folds. This approach provides unique insights into the directional bias of tertiary contact establishment that a traditional symmetric matrix would obscure, a directional effect that is strongly supported by previous protein folding literature [43,44]. We also identified a privileged interaction motif that could help us to design a privileged scaffold. This is a core chemical structure that targets a specific, recurring contact pattern found throughout an entire structural class [80,81].

Furthermore, we found that the contact frequencies of several residue pairs and their normalized values are highly correlated across the four structural classes. Therefore, we can improve fragment-based drug discovery (FBDD) by including fragments that can coordinate with these specific residues [82]. These contacts likely represent the fundamental physics of protein folding, namely hydrophobic collapse and van der Waals packing. These processes are essential for any stable fold, regardless of the secondary structure composition [49,83]. In the context of drug design, these stability hubs represent the most reliable regions for ligand anchoring, as their structural integrity is prioritized by evolution [84]. We also find that around 20% of other contacts exhibit the same spectrum of stability, ranging from moderate to very weak correlation. We therefore consider these contacts to exhibit a fractal stability spectrum ranging from very strong to very weak within the hydrolase superfamily. Rather than being isolated islands of stability, structural classes appear to be part of an interconnected stability landscape. Our results are supported by previous research demonstrating that enzymes are not uniformly stable. Instead, they are organized in concentric layers around the active site, alternating between ‘weak’ and ‘strong’, revealing an oscillatory pattern in which flexible, unstable active site residues are supported by a stable first shell of residues [85].

The consistency of this divergence pattern across 475 structures suggests that evolution modifies a specific, predictable subset of residue–residue interactions when switching fold from a helix bundle to a beta barrel. This scale-invariant divergence provides a roadmap for bio-isosteric drug design, whereby a ligand can be modified to remain stable when interacting with different yet statistically related protein targets [86].

In Stage 2, we uncover universal anchors by applying PCCs of RRCFM and NRRCFM among the four structural classes (Figure 6A–C and Figure 7A–C and Supplementary Figures S7–S12). Around 50% of the conserved interactions represent the evolutionary hardware that all stable proteins share. Therefore, we discovered that the inter-class (Stage 2) correlations mirror the distribution of the intra-class (Stage 1) correlations in a sort of universal hierarchy of structural stability. Our results reveal a dual-layered structural logic: while internal fold determinism exhibits a high-symmetry core, the transition between structural classes follows a UDP. This pattern remains similar across diverse inter-class comparisons, suggesting that the redistribution of long-range (∣i − j∣ ≥ 6) contacts is governed by a fixed statistical template, rather than by stochastic variation.

This study provides comprehensive statistical validation of a universal interaction grammar (UIG) that governs the protein landscape. Previous research has emphasized that the physical laws of folding are universal [87]. Other studies have shown that long-range contacts, which slow folding, also restrict native flexibility, identifying a unifying physical principle that governs protein function across evolution [88]. By analyzing 475 non-redundant SCOP archetypes, we have demonstrated that the grammar of protein stability is topologically invariant. The observation of statistical indistinguishability and a near-perfect linear relationship in interaction vectors across all structural classes shifts the focus from fold-specific geometry to universal chemical principles. Our findings reveal that the proteome relies on a conserved set of high-frequency anchors to maintain structural energetic stability, regardless of the surrounding secondary structure.

We have developed a framework to enhance fold-agnostic pharmacophore map-ping, which is important for medicinal chemistry [89,90,91], by demonstrating that the energetic benefits of chemical interactions are statistically similar across different folds (Figure 8). This methodology enables rational scaffold hopping and lead optimization without the need for 3D structural alignment. Our findings suggest that a ligand’s affinity is driven more by its adherence to the UG of the proteome than by its complementarity to a specific, unique topological shape. In Figure 9, the identification of a discrete multi-pocket arrangement provides direct mathematical and spatial evidence of compartmentalized stabilizing forces operating within the ABHD6 core. The invariance of these high Z-scores suggests that these contacts act as a physical universal grammar essential for maintaining the structural viability and catalytic pocket geometry of this class of enzymes.

The practical utility of this study lies in the transition from geometric-based modelling to grammar-based mapping. By demonstrating that interaction vectors are highly correlated across classes, we provide a mathematical justification for scaffold hopping without structural superposition. Medicinal chemists can now prioritize universal anchors (Z > 1.96) to ensure that a lead compound satisfies the fundamental grammar of the proteome. This increases the likelihood of cross-class binding and reducing the off-target uncertainty typically associated with non-homologous proteins.

In addition, the discovery of this invariant grammar opens several new avenues for research. Firstly, integrating this universal statistical reference into generative artificial intelligence (AI) models could streamline the de novo design of proteins and ligands by ensuring that they satisfy grammar-correct constraints. Secondly, further study is required to determine whether this grammar evolved due to the specific chemistry of the 20 standard amino acids or whether it represents a deeper physical law of molecular self-assembly. Thirdly, understanding the universal anchors could allow us to predict cross-reactivity and off-target binding more accurately in proteins that share similar grammar scores despite having zero structural homology. The existence of a UIG suggests that the grammar of protein stability may have been established early in prebiotic evolution, before the diversification of complex folds. The high frequency and invariant Z-scores of specific anchors represent the most efficient solutions to the problem of protein stabilization. Since then, evolution has copy-pasted this grammar into every known architecture, from the simplest helical bundles to the most complex beta barrels.

Furthermore, scientists had long believed that each protein fold had its own unique set of rules for maintaining stability. However, our study of 475 diverse protein structures proves otherwise. We discovered a UIG, a hidden statistical law that remains approximately the same across all protein shapes, especially in the hydrolase superfamily. Regardless of whether a protein looks like a spiral or a sheet, we found that it uses the same chemical grammar to hold itself together, with nearly identical mathematical precision. This discovery allows drug designers to scaffold hop, creating new medicines for different diseases by following these universal rules rather than being limited by the specific 3D shape of a single protein. The high correlation of contact frequencies observed between structural classes explains the success of privileged scaffolds in medicinal chemistry [92,93]. Our data suggest that these scaffolds are not merely promiscuous, but target highly conserved contact networks that are topologically invariant across different structural classes. Finally, as we summarize in Table 1, a quantitative benchmark highlights the multi-layered analytical capabilities of our descriptive topological framework against prior structural paradigms. At the macro-level, flattening the contact matrices into 1D vectors to compute cross-class covariance reveals an exceptionally high correlation ranging from 0.92 to 0.99 (*p* < 0.001). This confirms that all fold classes obey a universal physical law of structural packing. However, at the micro-level, when correlating the upper and lower triangles to map directional constraints, a profound scaling law emerges. Within the same structural class (intra-class), approximately 80% of contacts maintain a very strong (*p* < 0.001) to strong correlation, indicating rigid, reciprocal stability hubs. Remarkably, when performing these triangle evaluations across different structural classes (inter-class), the correlation profile does not collapse into random noise. Instead, every inter-class comparison consistently maintains approximately the exact same continuous mathematical spectrum, stretching from very strong positive correlations to definitive negative values. The defining topological distinction between the different structural folds lies exclusively in the shifting percentage distribution within this universal spectrum, where very strong (*p* < 0.001) to strong correlations encompass an average of approximately 50% of the contacts, depending on the specific class pairing. This combination of a shared, invariant correlation spectrum and fold-specific percentage modulations provides direct mathematical evidence of statistical self-similarity and scale invariance, fully validating classical protein mass fractal models. This demonstrates that, while all proteins utilize a single, universal geometric distribution pattern to govern the structural energetic stability of the backbone, individual fold architectures modulate the internal density weights to achieve their distinct tertiary scaffolds [46]. In conclusion, the UIG provides a robust mathematical foundation that simplifies the complexity of the proteome. This offers a clear path forward for designing more resilient therapeutics that leverage the fundamental, invariant laws of protein stability.

Although our coarse-grained network framework provides a robust topological roadmap of structural stability, several methodological limitations must be explicitly addressed. First, our baseline dataset was strictly restricted to high-resolution X-ray crystallography structures, entirely excluding alternative experimental approaches such as cryogenic electron microscopy (cryo-EM) or nuclear magnetic resonance (NMR) spectroscopy. While this constraint ensures maximum coordinate precision for the strict open interval of 3.0 to 5.0 Å, it may introduce resolution-specific biases into the baseline distributions. Second, our analysis evaluated exclusively the first subunit of each multi-chain protein. Although this standardization successfully isolates single-chain configurations and eliminates oligomeric assembly artifacts, it inherently omits the topological contribution of quaternary interfaces and inter-subunit stabilization anchors. Third, the selected multi-protein benchmark exhibits a pronounced structural bias toward the hydrolase enzyme family, which is overrepresented in the Protein Data Bank (PDB). Consequently, the contact frequencies and class-specific Z-score calibrations must be generalized with caution when applied to non-enzymatic folds or highly flexible transport proteins. Finally, our residue interaction networks are derived from static PDB coordinates, capturing a single spatial snapshot of the native state. This static abstraction cannot explicitly account for structural dynamics, conformational plasticity, or thermal fluctuations. Future iterations of this model will expand the baseline repository to include cryo-EM ensembles and molecular dynamics trajectories to reconcile these topological constraints with protein motion.

## 5. Conclusions

In this study, we have moved beyond traditional secondary structure analysis to define the mathematical grammar of the tertiary protein architecture of the hydrolase superfamily. By implementing a strict ∣i − j∣ ≥ 6 filter between only-C_α_, we isolate the residues that are far apart in sequence but close in 3D space (3–5 Å). We identify the non-local interactions that dictate global stability and determinism, rather than local folding rules. By applying multi-stage PC analysis, our results demonstrate dual-layered stability logic.

At the macro-level, the high PCCs of contact vectors, ranging from 0.99 to 0.92, reveal the UG of high-frequency structural anchors among the four structural classes. However, at the micro-level, using double-stage PCCs of RRCFM and NRRCFM, in Stage 1 the intra-class PCC of contact matrices achieve around 80% of contact, ranging from very strong to strong correlation between upper and lower diagonals, uncovering also the UG of high-frequency structural anchors. In Stage 2, the inter-class PCCs of RRCFM and NRRCFM contact matrices achieve around 50% of contact, ranging from very strong to strong correlation between upper and lower diagonals, discovering UGS and around 50% UDP with positive and negative correlations. Therefore, we uncover a spectrum of stability in both stages, related to different percentages of correlation in Stages 1 and 2. We consider these stages to have a fractal nature in terms of stability within the hydrolase superfamily.

This suggests that, while the global scaffold is conserved, the fine-tuned directional symmetry provides the architectural specificity necessary to distinguish between enzymatic folds. For the field of drug discovery, these findings provide a quantitative framework to identify stability hubs, defined as regions of high determinism that offer the most reliable targets for ligand binding, while utilizing conserved fingerprints to ensure class-specific drug selectivity and offer a precise mechanism for reducing off-target risks in drug design.

## Figures and Tables

**Table 1:** Quantitative correlation benchmarks and topological comparisons against structural paradigms.

Model	AlphaFold (AF2)	Fractal Symmetry	Our Model (Macro-Level) Inter-Class	Our Model (Micro-Level) Intra-Class	Our Model (Micro-Level) Inter-Class
Representation	All-atom/Deep Learning	Coarse-grained/All-atom	Coarse-grained (Cα)/Pearson Correlation	Coarse-grained (Cα)/Pearson Correlation	Coarse-grained (Cα)/Pearson Correlation
Analysis Level	Predictive/Deep Learning Model	Empirical/Power-Law Regression Fit	Analytical/Vectorized Inter-class Matrices	Analytical/Triangle Asymmetry (Same Class)	Analytical/Triangle Asymmetry (Across Classes)
Target Metric	Global coordinate generation and distance optimization	Spatial self-similarity and packing density scaling	Universal Structural Grammar	Universal Structural Grammar	Fold-specific anisotropy and invariant spectra
Correlation range	0.80–0.90 (Statistical Proxy of local core)	0.45–0.65 (Power-law linearity proxy)	0.92–0.99	~80% (0.60–0.99) approx 20% (0–0.59)	~50% (0.60–0.99) approx 50% (≤0.59)
Statistical Confidence	Variable / Not explicitly linear	Highly dependent on cutoff windows	≤0.001(Very strong)	≤0.001 (Very strong)	≤0.001 (Very strong)
Primary Biophysical insight	High 3D coordinate accuracy, lacks explicit chemical rules [16]	Proves the protein core is an anisotropic, porous medium rather than a homogeneous 3D solid [48].	Proves all structural folds converge upon invariant physical rules of space-packing, representing a shared law.	Captures highly conserved core anchoring networks (~80%) balanced by a flexible topological envelope (~20%).	Maps the distinct structural fingerprints and directional variations across different fold types.

## Data Availability

The data that supports the findings of this study are available within the article, Supplementary Materials, and https://github.com/FatinNasr/USGPearsonCorrelationAnalys (accessed on 3 June 2026).

## Supplementary Materials

The following supporting information can be downloaded at: https://www.mdpi.com/article/10.3390/pharmaceutics18060728/s1, Figure S1: Distribution of analyzed PDB entries across the four major structural classes. Table S1: Amino acid residue–residue contact frequencies in the all-α class; Table S2: Amino acids residue–residue contact frequencies in the all-β class; Table S3: Amino acid residue–residue contact frequencies in the α/β class; Table S4: Amino acid residue–residue contacts frequencies in the α+β class; Table S5: Z-score normalization of intra-class residue–residue contact frequencies in the all-α class; Table S6: Z-score normalization of intra-class residue–residue contact frequencies in the all-β class; Table S7: Z-score normalization of intra-class residue–residue contact frequencies in the α/β class; Table S8: Z-score normalization of intra-class residue–residue contact frequencies in the α+β class. Figure S2: Median distribution and IQR overlap statistics of amino acid contact profiles under universal space-packing constraints. Figure S3: Pearson correlation of raw and normalized intra-class residue contact frequencies in the all-α class. Figure S4: Pearson correlation of raw and normalized intra-class residue contact frequencies in the all-β class. Figure S5: Pearson correlation of raw and normalized intra-class residue contact frequencies in the α/β class. Figure S6: Pearson correlation of raw and normalized intra-class residue contact frequencies in the α+β class. Figure S7: Pearson correlation of raw and normalized inter-class residue contact frequencies between the all-α and all-β structural classes. Figure S8: Pearson correlation of raw and normalized inter-class residue contact frequencies between the all-α and α/β structural classes. Figure S9: Pearson correlation of raw and normalized inter-class residue contact frequencies between the all-α and α+β structural classes. Figure S10: Pearson correlation of raw and normalized inter-class residue contact frequencies between the all-β and α/β structural classes. Figure S11: Pearson correlation of raw and normalized inter-class residue contact frequencies between the all-β and α+β structural classes. Figure S12: Pearson correlation of raw and normalized inter-class residue contact frequencies between the α/β and α+β structural classes.
