# Supplementary material for: A Universal Structural Grammar in Enzyme Fold for Predicting Drug Target Stability: Deciphering Directional Scaffolding via Multi-Stage Pearson Correlation of Asymmetric Contact Matrices
Source: Pharmaceutics. 2026 Jun 12;18(6):728. doi: 10.3390/pharmaceutics18060728 (PMC13306498; doi:10.3390/pharmaceutics18060728)
Supplement: Supplementary file 1 [file pharmaceutics-18-00728-s001.zip › pharmaceutics-4323236-supplementary.pdf]

SUPPORTING INFORMATION

A Universal Structural Grammar in Enzyme Fold for Predicting Drug Target Stability: Deciphering Directional Scaffolding via Multi-Stage Pearson Correlation of Asymmetric Contact Matrices

Fatin Jannus * and Hilario Ramírez-Rodrigo

Department of Biochemistry and Molecular Biology I, Faculty of Sciences, University of Granada, Av. Fuentenueva, 18071 Granada, Spain
* Correspondence: fanjan@outlook.es

Index:

1. Dataset Curation and Structural Classification of Hydrolase Proteins

Figure S1: Distribution of analyzed PDB entries across the four major structural classes.

2. Amino Acid Residue-Residue Contacts Frequencies in Structural Classes of Hydrolase Proteins

Table S1: Amino acid residue-residue contacts frequencies in all-α class.

Table S2: Amino acids residue-residue contacts frequencies in all-β class.

Table S3: Amino acids residue-residue contacts frequencies in α/β class.

Table S4: Amino acids residue-residue contacts frequencies in α+β class.

3. Z-score Normalization of Intra-Class Residue-Residue Contact Frequencies in Structural Classes of Hydrolase Proteins

Table S5: Z-score normalization of intra-class residue-residue contact frequencies in all-α class.

Table S6: Z-score normalization of intra-class residue-residue contact frequencies in all-β class.

Table S7: Z-score normalization of intra-class residue-residue contact frequencies in α/β class.

Table S8: Z-score normalization of intra-class residue-residue contact frequencies in α+β class.

4. Quantitative Analysis of Residue-Specific Contact Frequencies across four Structural Classes of Hydrolase Proteins

Figure S2: Median distribution and IQR overlap statistics of amino acid contact profiles under universal space-packing constraints.

5. Pearson Correlation of Raw and Normalized Intra-Class Residue Contact Frequencies in Structural Classes of Hydrolase Proteins

Figure S3: Pearson correlation of raw and normalized intra-class residue contact frequencies in all-α class.

Figure S4: Pearson correlation of raw and normalized intra-class residue contact frequencies in all-β class.

Figure S5: Pearson correlation of raw and normalized intra-class residue contact frequencies in α/β class.

Figure S6: Pearson correlation of raw and normalized intra-class residue contact frequencies in α+β class.

6. Pearson Correlation of Raw and Normalized Inter-Class Residue Contact Frequencies between Structural Classes of Hydrolase Proteins

Figure S7: Pearson correlation of raw and normalized inter-class residue contact frequencies between structural classes all-α and all-β.

Figure S8: Pearson correlation of raw and normalized inter-class residue contact frequencies between structural classes all-α and α/β.

Figure S9: Pearson correlation of raw and normalized inter-class residue contact frequencies between structural classes all-α and α+β.

Figure S10: Pearson correlation of raw and normalized inter-class residue contact frequencies between structural classes all-β and α/β.

Figure S11: Pearson correlation of raw and normalized inter-class residue contact frequencies between structural classes all-β and α+β.

Figure S12: Pearson correlation of raw and normalized inter-class residue contact frequencies between structural classes α/β and α+β.

1. Dataset Curation and Structural Classification of Hydrolase Proteins

```
All-alpha PDB proteins:

  "1a17.pdb" "1a5t.pdb" "1a62.pdb" "1ad6.pdb" "1af7.pdb" "1ah7.pdb"
"1ak0.pdb" "1alu.pdb" "1aoa.pdb" "1aqe.pdb" "1ax8.pdb" "1axi.pdb"
  "1b0n.pdb" "1b0x.pdb" "1b3q.pdb" "1b4a.pdb" "1b4f.pdb" "1b67.pdb"
"1b6a.pdb" "1b79.pdb" "1b7v.pdb" "1b89.pdb" "1b8x.pdb" "1b9m.pdb"
  "1baj.pdb" "1baz.pdb" "1bea.pdb" "1bg5.pdb" "1bg8.pdb" "1bgf.pdb"
"1bh9.pdb" "1bhd.pdb" "1bja.pdb" "1bkr.pdb" "1bm9.pdb" "1bmt.pdb"
  "1bpo.pdb" "1bvb.pdb" "1bvp.pdb" "1bz4.pdb" "1bou.pdb" "1c1k.pdb"
"1c3c.pdb" "1c52.pdb" "1ccd.pdb" "1cd9.pdb" "1ci4.pdb" "1cmc.pdb"
  "1cqx.pdb" "1cuk.pdb" "1cun.pdb" "1cy5.pdb" "1d2z.pdb" "1d8l.pdb"
"1dbh.pdb" "1dk8.pdb" "1dnu.pdb" "1dov.pdb" "1dow.pdb" "1dvk.pdb"
  "1dvo.pdb" "1dw0.pdb" "1dwk.pdb" "1dxs.pdb" "1e6i.pdb" "1e7l.pdb"
"1e85.pdb" "1eak.pdb" "1ecm.pdb" "1ed1.pdb" "1eex.pdb" "1eg3.pdb"
  "1eh6.pdb" "1ei7.pdb" "1elk.pdb" "1elw.pdb" "1em9.pdb" "1evy.pdb"
"1exn.pdb" "1eyh.pdb" "1eyv.pdb" "1ez3.pdb" "1f0y.pdb" "1f1e.pdb"
  "1f1m.pdb" "1f7c.pdb" "1fc3.pdb" "1fch.pdb" "1fio.pdb" "1fip.pdb"
"1fkm.pdb" "1fnn.pdb" "1fp2.pdb" "1fp3.pdb" "1fpo.pdb" "1fse.pdb"
  "1fx7.pdb" "1fyh.pdb" "1g2y.pdb" "1g8e.pdb" "1gak.pdb" "1gkm.pdb"
"1go3.pdb" "1gp7.pdb" "1gpj.pdb" "1gs9.pdb" "1gux.pdb" "1gv2.pdb"
  "1gvd.pdb" "1gvj.pdb" "1gvn.pdb" "1gxm.pdb" "1gyg.pdb" "1h12.pdb"
"1h1o.pdb" "1h21.pdb" "1h3l.pdb" "1h3o.pdb"
```

```
All-beta PDB proteins:

  "1a12.pdb" "1a1x.pdb" "1a62.pdb" "1a8d.pdb" "1agj.pdb" "1ahs.pdb"
"1air.pdb" "1ajk.pdb" "1ajo.pdb" "1aly.pdb" "1am2.pdb" "1aol.pdb"
  "1at0.pdb" "1auy.pdb" "1axi.pdb" "1axk.pdb" "1b12.pdb" "1b35.pdb"
"1b3q.pdb" "1b5f.pdb" "1b8a.pdb" "1b9m.pdb" "1bai.pdb" "1bhe.pdb"
  "1bkb.pdb" "1bn8.pdb" "1bpl.pdb" "1bpo.pdb" "1bqu.pdb" "1btk.pdb"
"1btn.pdb" "1bu8.pdb" "1bvp.pdb" "1c4q.pdb" "1c5e.pdb" "1c8n.pdb"
  "1c9o.pdb" "1ccz.pdb" "1cd9.pdb" "1cdc.pdb" "1cfb.pdb" "1ckm.pdb"
"1cq3.pdb" "1cqx.pdb" "1cr5.pdb" "1cru.pdb" "1cs6.pdb" "1cuk.pdb"
  "1cwv.pdb" "1d2o.pdb" "1d2p.pdb" "1d2s.pdb" "1d3b.pdb" "1d8l.pdb"
"1dbh.pdb" "1dd1.pdb" "1ddg.pdb" "1dfa.pdb" "1dgw.pdb" "1dle.pdb"
  "1dnl.pdb" "1dq3.pdb" "1dqg.pdb" "1dyo.pdb" "1dyp.pdb" "1e0b.pdb"
"1e2w.pdb" "1e6a.pdb" "1e7n.pdb" "1eaj.pdb" "1eaq.pdb" "1ear.pdb"
  "1eaz.pdb" "1eg3.pdb" "1ei5.pdb" "1eje.pdb" "1ejf.pdb" "1ep0.pdb"
"1epa.pdb" "1epf.pdb" "1ern.pdb" "1ex4.pdb" "1ezg.pdb" "1f0l.pdb"
  "1f2n.pdb" "1f35.pdb" "1f39.pdb" "1f3u.pdb" "1f56.pdb" "1f7d.pdb"
"1f86.pdb" "1f8e.pdb" "1fao.pdb" "1fgu.pdb" "1fgy.pdb" "1fhg.pdb"
  "1fl0.pdb" "1flk.pdb" "1fmk.pdb" "1fnf.pdb" "1fqt.pdb" "1fwy.pdb"
"1fx7.pdb" "1fyh.pdb" "1g1c.pdb" "1g2b.pdb" "1g31.pdb" "1g3p.pdb"
  "1fi2.pdb" "1fjr.pdb" "1g6g.pdb" "1g8f.pdb" "1gcy.pdb" "1gff.pdb"
```

```
Alpha/Beta PDB proteins:

  "1a2o.pdb" "1a76.pdb" "1aq0.pdb" "1avq.pdb" "1b7e.pdb" "1bif.pdb"
"1bn7.pdb" "1bpl.pdb" "1bqc.pdb" "1bu8.pdb" "1c7j.pdb" "1c8x.pdb"
  "1ceo.pdb" "1cex.pdb" "1cg2.pdb" "1chd.pdb" "1cpb.pdb" "1cr1.pdb"
"1cvl.pdb" "1d1q.pdb" "1d2n.pdb" "1d5r.pdb" "1dkl.pdb" "1e2s.pdb"
  "1edq.pdb" "1ehy.pdb" "1ei9.pdb" "1eke.pdb" "1eok.pdb" "1es9.pdb"
"1fcq.pdb" "1fj2.pdb" "1fob.pdb" "1fpz.pdb" "1fqo.pdb" "1fuk.pdb"
  "1fye.pdb" "1g2i.pdb" "1g66.pdb" "1g8m.pdb" "1ga6.pdb" "1gcy.pdb"
"1gyt.pdb" "1gzj.pdb" "1h09.pdb" "1h65.pdb" "1hdh.pdb" "1hjr.pdb"
  "1hl9.pdb" "1ht6.pdb" "1i24.pdb" "1i6w.pdb" "1ihp.pdb" "1ihu.pdb"
"1ilw.pdb" "1in4.pdb" "1ir6.pdb" "1itu.pdb" "1itx.pdb" "1iu8.pdb"
  "1ivy.pdb" "1j1i.pdb" "1j5x.pdb" "1j97.pdb" "1j9l.pdb" "1jak.pdb"
"1jeq.pdb" "1jfx.pdb" "1jjf.pdb" "1jl1.pdb" "1jrl.pdb" "1k0z.pdb"
  "1k1e.pdb" "1k20.pdb" "1k7c.pdb" "1k7k.pdb" "1kcf.pdb" "1knv.pdb"
"1ko7.pdb" "1kp0.pdb" "1kxq.pdb" "1l6r.pdb" "1l7a.pdb" "1lam.pdb"
  "1li4.pdb" "1lv7.pdb" "1lw3.pdb" "1lyv.pdb" "1m0d.pdb" "1m22.pdb"
"1m4l.pdb" "1m65.pdb" "1mkp.pdb" "1mug.pdb" "1mxg.pdb" "1na6.pdb"
  "1nd6.pdb" "1nf9.pdb" "1njr.pdb" "1nms.pdb" "1nnl.pdb" "1nof.pdb"
"1nu0.pdb" "1ny1.pdb" "1o6i.pdb" "1o7a.pdb" "1o7d.pdb" "1o7j.pdb"
  "1o8u.pdb" "1obb.pdb" "1oc7.pdb" "1ofh.pdb" "1ogs.pdb" "1ohe.pdb"
"1okj.pdb" "1onx.pdb" "1ox7.pdb"
```

```
Alpha+Beta PDB proteins:

  "1a2p.pdb" "1a6f.pdb" "1a6q.pdb" "1ae9.pdb" "1af5.pdb" "1aqz.pdb"
"1aui.pdb" "1b24.pdb" "1b6a.pdb" "1bol.pdb" "1bvq.pdb" "1c7k.pdb"
  "1cg2.pdb" "1cs8.pdb" "1cse.pdb" "1d8i.pdb" "1deu.pdb" "1dfa.pdb"
"1dki.pdb" "1dxj.pdb" "1dy5.pdb" "1e1h.pdb" "1eak.pdb" "1h70.pdb"
  "1edq.pdb" "1fbx.pdb" "1fit.pdb" "1g0s.pdb" "1g3k.pdb" "1g5b.pdb"
"1gbs.pdb" "1hd7.pdb" "1hp1.pdb" "1hr6.pdb" "1huf.pdb" "1i8v.pdb"
  "1id0.pdb" "1ii7.pdb" "1iqq.pdb" "1itx.pdb" "1jak.pdb" "1jh6.pdb"
"1k07.pdb" "1k28.pdb" "1k2x.pdb" "1k9x.pdb" "1kap.pdb" "1khq.pdb"
  "1ko9.pdb" "1kp0.pdb" "1kpf.pdb" "1kug.pdb" "1l4d.pdb" "1lbu.pdb"
"1lml.pdb" "1lqv.pdb" "1mc0.pdb" "1mhw.pdb" "1mk0.pdb" "1mpg.pdb"
  "1mqe.pdb" "1mu7.pdb" "1nln.pdb" "1nqz.pdb" "1nww.pdb" "1nz0.pdb"
"1o0w.pdb" "1o6i.pdb" "1o7a.pdb" "1obb.pdb" "1or0.pdb" "1ouo.pdb"
  "1p42.pdb" "1p4d.pdb" "1pxv.pdb" "1q32.pdb" "1q3i.pdb" "1q3q.pdb"
"1q4u.pdb" "1qcs.pdb" "1qgi.pdb" "1qh5.pdb" "1qjj.pdb" "1qvj.pdb"
  "1r44.pdb" "1r9w.pdb" "1rbd.pdb" "1rl0.pdb" "1rre.pdb" "1rya.pdb"
"1s2j.pdb" "1s3m.pdb" "1s3s.pdb" "1s67.pdb" "1s95.pdb" "1s9r.pdb"
  "1sjy.pdb" "1slm.pdb" "1smn.pdb" "1sp4.pdb" "1squ.pdb" "1sx7.pdb"
"1sxr.pdb" "1t3c.pdb" "1t4o.pdb" "1tff.pdb" "1th0.pdb" "1txo.pdb"
  "1tzp.pdb" "1u20.pdb" "1u4g.pdb" "1u8x.pdb" "1uf5.pdb" "1ukf.pdb"
"1umg.pdb" "1urr.pdb" "1ute.pdb" "1v8y.pdb" "1v9y.pdb" "1vcd.pdb"
  "1vf8.pdb" "1vh5.pdb" "1vj7.pdb" "1vjv.pdb" "1vk6.pdb" "1vpm.pdb"
```

Figure S1: Distribution of analyzed PDB entries across the four major structural classes.

2. Amino acid residue-residue contacts frequencies in structural classes of hydrolase proteins

All-α Class	A	C	D	E	F	G	H	I	K	L	M	N	P	Q	R	S	T	V	W	Y
**A**	326	47	114	107	232	121	78	299	122	500	102	67	77	94	169	124	152	263	78	222
**C**	65	92	19	35	59	37	25	75	55	119	24	25	25	12	32	32	39	64	18	43
**D**	86	19	93	80	84	78	76	84	177	138	41	79	62	63	229	88	106	113	36	133
**E**	103	32	74	88	115	77	72	131	213	247	53	95	75	97	249	113	101	143	42	155
**F**	229	70	119	102	290	124	57	299	111	533	117	75	105	103	155	124	131	274	111	247
**G**	168	30	122	116	138	144	61	146	109	219	60	80	88	85	130	86	103	154	51	153
**H**	84	20	68	68	72	54	58	66	53	121	43	43	52	56	77	59	54	90	24	77
**I**	320	58	126	159	289	143	76	472	157	842	150	100	119	117	175	128	190	446	85	243
**K**	121	34	180	224	124	110	31	133	98	250	58	103	82	83	107	105	117	143	54	174
**L**	534	134	141	236	532	195	114	764	237	1452	260	141	185	184	313	221	269	692	154	442
**M**	122	35	52	57	110	60	28	137	58	292	62	51	61	53	73	61	63	151	39	104
**N**	102	23	71	94	69	87	34	86	105	123	30	83	54	74	132	89	86	68	41	102
**P**	107	21	75	88	97	90	53	119	78	180	55	63	66	47	117	70	76	83	44	153
**Q**	103	25	67	95	94	87	37	79	72	187	42	67	73	71	121	92	82	96	30	104
**R**	154	39	211	279	143	123	71	167	96	302	71	110	89	103	153	133	130	158	51	162
**S**	131	45	98	95	122	104	56	118	106	199	65	82	73	69	116	124	85	131	59	134
**T**	154	41	95	104	118	115	50	140	96	229	52	70	81	76	141	93	100	165	61	126
**V**	349	70	94	142	281	135	77	384	150	716	135	81	118	107	168	141	189	436	79	215
**W**	96	25	71	49	124	81	54	104	52	216	49	38	65	58	75	52	56	109	35	108
**Y**	186	56	121	125	182	129	87	208	167	367	87	90	107	88	152	108	121	202	86	177

Table S1: Amino acid residue-residue contacts frequencies in all-α class of hydrolase proteins (118 PDB).

All-β Class	A	C	D	E	F	G	H	I	K	L	M	N	P	Q	R	S	T	V	W	Y
**A**	275	58	134	144	265	201	84	338	176	501	101	142	150	134	182	199	239	410	137	216
**C**	84	157	48	33	93	69	35	95	63	139	42	49	55	38	56	77	78	117	47	78
**D**	176	50	144	117	158	176	123	185	276	233	62	232	129	113	302	167	190	207	96	234
**E**	149	48	97	134	160	157	82	234	280	291	59	165	128	101	297	144	220	257	97	224
**F**	262	122	164	165	418	181	115	489	210	660	154	152	199	145	216	225	217	496	185	335
**G**	211	64	171	172	252	237	102	256	191	333	100	218	177	150	221	239	233	328	134	223
**H**	106	36	107	80	94	90	77	124	82	144	61	98	80	67	111	119	97	140	57	129
**I**	364	96	175	219	487	229	130	711	205	863	174	179	184	166	206	243	302	754	170	351
**K**	165	56	253	328	174	180	51	214	154	280	67	164	126	110	140	209	223	274	91	227
**L**	481	133	204	241	618	283	137	922	287	1313	222	211	275	235	371	347	374	970	243	526
**M**	108	42	67	69	147	72	52	165	57	245	66	61	65	42	89	79	79	188	54	109
**N**	152	51	168	127	137	190	66	151	189	207	30	209	129	124	194	202	228	189	105	188
**P**	173	59	125	143	177	183	72	205	134	249	82	153	165	82	192	188	176	258	119	239
**Q**	109	39	106	105	130	131	70	126	132	211	39	120	107	100	114	136	107	193	57	139
**R**	224	77	293	308	222	209	91	250	137	301	70	172	148	142	185	220	190	306	130	236
**S**	195	68	186	194	229	208	115	229	194	335	78	178	171	130	222	254	268	305	121	221
**T**	266	63	193	227	258	252	110	348	228	414	84	242	194	169	235	280	358	400	147	256
**V**	441	133	200	251	492	298	129	745	281	960	166	213	246	214	301	312	435	893	194	396
**W**	138	63	102	103	167	117	51	224	98	270	71	95	123	95	124	134	115	246	92	167
**Y**	220	82	184	168	260	211	104	337	253	460	123	190	213	136	233	218	233	376	166	273

Table S2: Amino acid residue-residue contacts frequencies in all-β class of hydrolase proteins (114 PDB).

α/β Class	A	C	D	E	F	G	H	I	K	L	M	N	P	Q	R	S	T	V	W	Y
A	519	81	234	223	444	372	179	552	232	895	198	223	282	183	347	277	331	699	218	384
C	105	127	55	56	92	66	55	112	53	167	39	37	51	40	77	84	71	136	35	97
D	252	51	237	191	240	299	193	214	318	282	98	229	160	145	436	260	244	235	152	346
E	216	35	154	154	210	199	163	219	318	304	82	184	163	126	424	214	202	239	141	288
F	455	109	209	218	582	252	179	641	214	981	218	210	248	149	285	269	266	665	215	422
G	447	96	281	239	361	417	169	320	235	529	165	300	236	198	378	341	403	430	181	416
H	134	37	184	177	184	141	113	143	92	255	74	107	124	79	138	148	146	181	103	159
I	621	99	241	269	687	368	139	1001	251	1453	290	201	278	184	315	269	368	972	237	500
K	260	43	363	307	225	256	66	208	147	344	64	178	153	126	190	209	209	260	105	283
L	931	187	312	389	970	455	261	1321	336	2239	348	314	409	328	524	427	557	1406	374	715
M	181	37	91	78	234	135	74	244	84	355	96	87	96	63	114	126	135	258	95	169
N	194	57	243	207	206	247	122	181	187	271	61	235	183	164	258	221	214	268	109	247
P	255	74	218	168	313	245	140	248	151	470	111	194	216	141	288	222	218	307	176	359
Q	157	45	131	114	157	161	92	145	121	278	65	142	140	107	220	181	159	195	106	203
R	341	59	464	505	285	337	170	296	185	484	104	241	252	191	316	284	283	329	161	322
S	310	83	291	235	291	323	176	275	206	462	128	237	208	177	319	254	268	337	153	269
T	384	82	261	213	342	327	173	380	206	535	147	252	237	184	264	279	297	466	170	325
V	681	119	285	282	696	378	203	931	303	1290	284	232	302	193	367	342	444	1110	255	499
W	203	47	140	147	255	143	80	233	94	381	89	114	131	110	156	158	123	255	139	240
Y	420	81	326	283	459	330	189	459	213	649	171	238	317	188	352	275	282	475	220	388

Table S3: Amino acid residue-residue contacts frequencies in α/β class of hydrolase proteins (117 PDB).

α+β Class	A	C	D	E	F	G	H	I	K	L	M	N	P	Q	R	S	T	V	W	Y
A	403	73	155	175	342	262	152	428	172	663	143	166	167	120	198	201	243	533	150	290
C	87	166	42	56	70	69	54	105	56	146	30	38	72	50	50	68	84	133	29	104
D	171	58	170	148	165	211	167	146	247	216	70	172	122	108	298	179	187	201	101	221
E	159	40	128	139	172	171	91	173	250	254	68	142	149	114	387	163	156	216	73	227
F	319	103	169	197	476	216	141	473	169	763	142	136	187	131	223	216	218	449	209	342
G	267	74	238	222	248	320	129	278	182	381	94	170	155	136	223	214	225	317	112	316
H	113	40	144	166	130	133	132	118	58	215	62	90	88	74	96	127	124	156	77	187
I	408	85	163	227	472	263	128	774	179	988	202	141	174	132	213	254	265	668	170	335
K	175	45	269	282	169	175	67	221	134	288	73	150	142	135	144	183	171	220	72	230
L	642	153	243	305	722	354	202	978	287	1605	296	229	294	213	366	312	387	985	269	539
M	139	38	72	87	175	90	42	170	53	255	87	73	84	51	91	64	83	195	72	114
N	126	37	142	150	166	182	107	135	146	201	43	153	105	109	169	155	150	183	94	197
P	159	50	113	151	159	160	96	150	121	296	58	104	134	81	161	134	128	185	111	241
Q	145	29	122	125	131	162	80	149	103	241	53	109	128	95	142	112	147	183	86	170
R	212	70	325	403	215	237	114	234	155	398	86	169	202	138	240	192	214	292	129	300
S	181	85	191	160	210	192	127	227	148	301	73	144	151	128	191	198	169	259	88	232
T	267	79	167	186	219	269	116	308	200	403	83	155	170	125	205	174	195	321	97	245
V	495	113	169	256	449	315	149	634	210	1027	192	175	249	163	250	258	302	805	161	412
W	131	40	99	82	156	147	41	153	64	280	58	68	119	61	104	98	82	168	71	144
Y	305	87	267	198	380	284	139	372	204	527	141	160	244	137	282	199	244	374	141	312

Table S4: Amino acid residue-residue contacts frequencies in α+β class protein of hydrolase proteins (126 PDB).

3. Z-score normalization of amino acid residue-residue contacts frequencies in structural classes of hydrolase proteins

	A	C	D	E	F	G	H	I	K	L	M	N	P	Q	R	S	T	V	W	Y
A	1,5977	-0,6461	-0,1073	-0,1636	0,8417	-0,051	-0,3968	1,3806	-0,0429	2,9971	-0,2038	-0,4853	-0,4048	-0,2681	0,3351	-0,0268	0,1983	1,0911	-0,3968	0,7613
C	-0,5013	-0,2842	-0,8713	-0,7426	-0,5496	-0,7265	-0,823	-0,4209	-0,5818	-0,0671	-0,8311	-0,823	-0,823	-0,9276	-0,7667	-0,7667	-0,7104	-0,5094	-0,8793	-0,6783
D	-0,3325	-0,8713	-0,2762	-0,3807	-0,3485	-0,3968	-0,4129	-0,3485	0,3994	0,0858	-0,6944	-0,3887	-0,5255	-0,5174	0,8176	-0,3164	-0,1716	-0,1153	-0,7346	0,0455
E	-0,1957	-0,7667	-0,429	-0,3164	-0,0992	-0,4048	-0,445	0,0295	0,6889	0,9624	-0,5979	-0,2601	-0,4209	-0,244	0,9785	-0,1153	-0,2118	0,126	-0,6863	0,2225
F	0,8176	-0,4611	-0,0671	-0,2038	1,3082	-0,0268	-0,5657	1,3806	-0,1314	3,2625	-0,0831	-0,4209	-0,1796	-0,1957	0,2225	-0,0268	0,0295	1,1795	-0,1314	0,9624
G	0,327	-0,7828	-0,0429	-0,0912	0,0858	0,134	-0,5335	0,1501	-0,1475	0,7372	-0,5416	-0,3807	-0,3164	-0,3405	0,0214	-0,3325	-0,1957	0,2144	-0,6139	0,2064
H	-0,3485	-0,8633	-0,4772	-0,4772	-0,445	-0,5898	-0,5576	-0,4933	-0,5979	-0,051	-0,6783	-0,6783	-0,6059	-0,5737	-0,4048	-0,5496	-0,5898	-0,3003	-0,8311	-0,4048
I	1,5495	-0,5576	-0,0108	0,2546	1,3002	0,126	-0,4129	2,7719	0,2386	5,7476	0,1823	-0,2199	-0,0671	-0,0831	0,3833	0,0053	0,504	2,5628	-0,3405	0,9302
K	-0,051	-0,7507	0,4235	0,7774	-0,0268	-0,1394	-0,7748	0,0455	-0,2359	0,9865	-0,5576	-0,1957	-0,3646	-0,3566	-0,1636	-0,1796	-0,0831	0,126	-0,5898	0,3753
L	3,2705	0,0536	0,1099	0,8739	3,2545	0,5442	-0,1073	5,1203	0,8819	10,6535	1,0669	0,1099	0,4637	0,4557	1,4932	0,7533	1,1393	4,5412	0,2144	2,5306
M	-0,0429	-0,7426	-0,6059	-0,5657	-0,1394	-0,5416	-0,7989	0,0777	-0,5576	1,3243	-0,5255	-0,6139	-0,5335	-0,5979	-0,437	-0,5335	-0,5174	0,1903	-0,7104	-0,1877
N	-0,2038	-0,8391	-0,4531	-0,2681	-0,4692	-0,3244	-0,7507	-0,3325	-0,1796	-0,0349	-0,7828	-0,3566	-0,5898	-0,429	0,0375	-0,3083	-0,3325	-0,4772	-0,6944	-0,2038
P	-0,1636	-0,8552	-0,4209	-0,3164	-0,244	-0,3003	-0,5979	-0,0671	-0,3968	0,4235	-0,5818	-0,5174	-0,4933	-0,6461	-0,0831	-0,4611	-0,4129	-0,3566	-0,6702	0,2064
Q	-0,1957	-0,823	-0,4853	-0,2601	-0,2681	-0,3244	-0,7265	-0,3887	-0,445	0,4798	-0,6863	-0,4853	-0,437	-0,4531	-0,051	-0,2842	-0,3646	-0,252	-0,7828	-0,1877
R	0,2144	-0,7104	0,6728	1,2197	0,126	-0,0349	-0,4531	0,319	-0,252	1,4047	-0,4531	-0,1394	-0,3083	-0,1957	0,2064	0,0455	0,0214	0,2466	-0,6139	0,2788
S	0,0295	-0,6622	-0,2359	-0,2601	-0,0429	-0,1877	-0,5737	-0,0751	-0,1716	0,5763	-0,5013	-0,3646	-0,437	-0,4692	-0,0912	-0,0268	-0,3405	0,0295	-0,5496	0,0536
T	0,2144	-0,6944	-0,2601	-0,1877	-0,0751	-0,0992	-0,622	0,1018	-0,252	0,8176	-0,6059	-0,4611	-0,3727	-0,4129	0,1099	-0,2762	-0,2199	0,3029	-0,5335	-0,0108
V	1,7827	-0,4611	-0,2681	0,1179	1,2358	0,0616	-0,4048	2,0642	0,1823	4,7343	0,0616	-0,3727	-0,0751	-0,1636	0,327	0,1099	0,4959	2,4824	-0,3887	0,705
W	-0,252	-0,823	-0,4531	-0,63	-0,0268	-0,3727	-0,5898	-0,1877	-0,6059	0,7131	-0,63	-0,7185	-0,5013	-0,5576	-0,4209	-0,6059	-0,5737	-0,1475	-0,7426	-0,1555
Y	0,4718	-0,5737	-0,051	-0,0188	0,4396	0,0134	-0,3244	0,6487	0,319	1,9275	-0,3244	-0,3003	-0,1636	-0,3164	0,1983	-0,1555	-0,051	0,6005	-0,3325	0,3994

Table S5: Z-score normalization of amino acid residue-residue contacts frequencies in all-α class (118 PDB).

	A	C	D	E	F	G	H	I	K	L	M	N	P	Q	R	S	T	V	W	Y
A	0,5294	-0,9317	-0,42	-0,3526	0,4621	0,0312	-0,7567	0,9536	-0,1372	2,0512	-0,6422	-0,3661	-0,3122	-0,42	-0,0968	0,0177	0,287	1,4384	-0,3998	0,1322
C	-0,7567	-0,2651	-0,9991	-1,1001	-0,6961	-0,8577	-1,0866	-0,6826	-0,8981	-0,3863	-1,0395	-0,9923	-0,9519	-1,0664	-0,9452	-0,8038	-0,7971	-0,5344	-1,0058	-0,7971
D	-0,1372	-0,9856	-0,3526	-0,5344	-0,2584	-0,1372	-0,494	-0,0766	0,5362	0,2466	-0,9048	0,2399	-0,4536	-0,5614	0,7112	-0,1978	-0,0429	0,0716	-0,6759	0,2534
E	-0,319	-0,9991	-0,6691	-0,42	-0,2449	-0,2651	-0,7701	0,2534	0,5631	0,6372	-0,925	-0,2112	-0,4604	-0,6422	0,6776	-0,3526	0,1591	0,4082	-0,6691	0,186
F	0,4419	-0,5008	-0,218	-0,2112	1,4923	-0,1035	-0,5479	1,9704	0,0918	3,1218	-0,2853	-0,2988	0,0177	-0,3459	0,1322	0,1928	0,1389	2,0175	-0,0766	0,9334
G	0,0985	-0,8913	-0,1708	-0,1641	0,3746	0,2736	-0,6355	0,4015	-0,0362	0,92	-0,6489	0,1456	-0,1304	-0,3122	0,1658	0,287	0,2466	0,8863	-0,42	0,1793
H	-0,6085	-1,0799	-0,6018	-0,7836	-0,6893	-0,7163	-0,8038	-0,4873	-0,7701	-0,3526	-0,9115	-0,6624	-0,7836	-0,8711	-0,5748	-0,521	-0,6691	-0,3796	-0,9385	-0,4536
I	1,1287	-0,6759	-0,1439	0,1524	1,9569	0,2197	-0,4469	3,4652	0,0581	4,4887	-0,1506	-0,117	-0,0833	-0,2045	0,0648	0,314	0,7112	3,7547	-0,1776	1,0412
K	-0,2112	-0,9452	0,3813	0,8863	-0,1506	-0,1102	-0,9789	0,1187	-0,2853	0,5631	-0,8711	-0,218	-0,4738	-0,5816	-0,3796	0,085	0,1793	0,5227	-0,7095	0,2062
L	1,9165	-0,4267	0,0514	0,3005	2,839	0,5833	-0,3998	4,886	0,6102	7,5187	0,1726	0,0985	0,5294	0,2601	1,1758	1,0142	1,196	5,2092	0,314	2,2195
M	-0,595	-1,0395	-0,8711	-0,8577	-0,3324	-0,8375	-0,9721	-0,2112	-0,9385	0,3274	-0,8779	-0,9115	-0,8846	-1,0395	-0,723	-0,7903	-0,7903	-0,0564	-0,9587	-0,5883
N	-0,2988	-0,9789	-0,191	-0,4671	-0,3998	-0,0429	-0,8779	-0,3055	-0,0496	0,0716	-1,1203	0,085	-0,4536	-0,4873	-0,016	0,0379	0,213	-0,0496	-0,6152	-0,0564
P	-0,1574	-0,925	-0,4806	-0,3594	-0,1304	-0,09	-0,8375	0,0581	-0,42	0,3544	-0,7701	-0,292	-0,2112	-0,7701	-0,0294	-0,0564	-0,1372	0,415	-0,521	0,287
Q	-0,5883	-1,0597	-0,6085	-0,6152	-0,4469	-0,4402	-0,8509	-0,4738	-0,4334	0,0985	-1,0597	-0,5142	-0,6018	-0,6489	-0,5546	-0,4065	-0,6018	-0,0227	-0,9385	-0,3863
R	0,186	-0,8038	0,6506	0,7516	0,1726	0,085	-0,7095	0,3611	-0,3998	0,7045	-0,8509	-0,1641	-0,3257	-0,3661	-0,0766	0,1591	-0,0429	0,7382	-0,4469	0,2668
S	-0,0092	-0,8644	-0,0698	-0,016	0,2197	0,0783	-0,5479	0,2197	-0,016	0,9334	-0,7971	-0,1237	-0,1708	-0,4469	0,1726	0,388	0,4823	0,7314	-0,5075	0,1658
T	0,4688	-0,8981	-0,0227	0,2062	0,415	0,3746	-0,5816	1,021	0,213	1,4654	-0,7567	0,3072	-0,016	-0,1843	0,2601	0,5631	1,0883	1,3711	-0,3324	0,4015
V	1,6472	-0,4267	0,0244	0,3678	1,9906	0,6843	-0,4536	3,6941	0,5698	5,1418	-0,2045	0,112	0,3342	0,1187	0,7045	0,7786	1,6068	4,6907	-0,016	1,3442
W	-0,393	-0,8981	-0,6355	-0,6287	-0,1978	-0,5344	-0,9789	0,186	-0,6624	0,4958	-0,8442	-0,6826	-0,494	-0,6826	-0,4873	-0,42	-0,5479	0,3342	-0,7028	-0,1978
Y	0,1591	-0,7701	-0,0833	-0,191	0,4284	0,0985	-0,622	0,9469	0,3813	1,7751	-0,494	-0,0429	0,112	-0,4065	0,2466	0,1456	0,2466	1,2095	-0,2045	0,516

Table S6: Z-score normalization of amino amino acids residue-residue contacts frequencies in all-β class (114 PDB).

	A	C	D	E	F	G	H	I	K	L	M	N	P	Q	R	S	T	V	W	Y
A	1,1271	-0,8393	-0,1524	-0,2018	0,7904	0,4671	-0,3993	1,2752	-0,1614	2,8151	-0,314	-0,2018	0,0631	-0,3814	0,3549	0,0406	0,2831	1,9352	-0,2242	0,521
C	-0,7316	-0,6328	-0,956	-0,9515	-0,7899	-0,9066	-0,956	-0,7001	-0,965	-0,4532	-1,0279	-1,0368	-0,974	-1,0234	-0,8573	-0,8258	-0,8842	-0,5924	-1,0458	-0,7675
D	-0,0716	-0,974	-0,1389	-0,3455	-0,1255	0,1394	-0,3365	-0,2422	0,2247	0,0631	-0,763	-0,1749	-0,4846	-0,552	0,7545	-0,0357	-0,1075	-0,1479	-0,5205	0,3504
E	-0,2332	-1,0458	-0,5116	-0,5116	-0,2602	-0,3095	-0,4712	-0,2197	0,2247	0,1619	-0,8348	-0,3769	-0,4712	-0,6373	0,7006	-0,2422	-0,2961	-0,13	-0,5699	0,09
F	0,8398	-0,7136	-0,2646	-0,2242	1,4099	-0,0716	-0,3993	1,6748	-0,2422	3,2012	-0,2242	-0,2602	-0,0896	-0,534	0,0766	0,0047	-0,0087	1,7826	-0,2377	0,6916
G	0,8039	-0,772	0,0586	-0,13	0,4178	0,6692	-0,4442	0,2337	-0,1479	1,172	-0,4622	0,1439	-0,1434	-0,314	0,4941	0,328	0,6063	0,7275	-0,3903	0,6647
H	-0,6014	-1,0368	-0,3769	-0,4083	-0,3769	-0,5699	-0,6956	-0,561	-0,7899	-0,0581	-0,8707	-0,7226	-0,6463	-0,8483	-0,5834	-0,5385	-0,5475	-0,3903	-0,7405	-0,4891
I	1,585	-0,7585	-0,121	0,0047	1,8813	0,4492	-0,5789	3,291	-0,0761	5,3203	0,099	-0,3006	0,0451	-0,3769	0,2112	0,0047	0,4492	3,1608	-0,1389	1,0418
K	-0,0357	-1,0099	0,4267	0,1753	-0,1928	-0,0536	-0,9066	-0,2691	-0,543	0,3414	-0,9156	-0,4038	-0,5161	-0,6373	-0,3499	-0,2646	-0,2646	-0,0357	-0,7316	0,0676
L	2,9768	-0,3634	0,1978	0,5435	3,1519	0,8398	-0,0312	4,7277	0,3055	8,849	0,3594	0,2068	0,6333	0,2696	1,1495	0,7141	1,2977	5,1093	0,4761	2,007
M	-0,3903	-1,0368	-0,7944	-0,8528	-0,1524	-0,5969	-0,8707	-0,1075	-0,8258	0,3908	-0,772	-0,8124	-0,772	-0,9201	-0,6911	-0,6373	-0,5969	-0,0447	-0,7764	-0,4442
N	-0,332	-0,947	-0,112	-0,2736	-0,2781	-0,094	-0,6552	-0,3903	-0,3634	0,0137	-0,9291	-0,1479	-0,3814	-0,4667	-0,0447	-0,2108	-0,2422	0,0002	-0,7136	-0,094
P	-0,0581	-0,8707	-0,2242	-0,4487	0,2023	-0,103	-0,5744	-0,0896	-0,525	0,9071	-0,7046	-0,332	-0,2332	-0,5699	0,09	-0,2063	-0,2242	0,1753	-0,4128	0,4088
Q	-0,4981	-1,0009	-0,6148	-0,6911	-0,4981	-0,4801	-0,7899	-0,552	-0,6597	0,0451	-0,9111	-0,5654	-0,5744	-0,7226	-0,2153	-0,3903	-0,4891	-0,3275	-0,7271	-0,2916
R	0,328	-0,9381	0,8802	1,0642	0,0766	0,31	-0,4397	0,1259	-0,3724	0,97	-0,736	-0,121	-0,0716	-0,3455	0,2157	0,0721	0,0676	0,2741	-0,4801	0,2427
S	0,1888	-0,8303	0,1035	-0,1479	0,1035	0,2472	-0,4128	0,0317	-0,2781	0,8712	-0,6283	-0,1389	-0,2691	-0,4083	0,2292	-0,0626	0,0002	0,31	-0,5161	0,0047
T	0,521	-0,8348	-0,0312	-0,2467	0,3325	0,2651	-0,4263	0,5031	-0,2781	1,1989	-0,543	-0,0716	-0,1389	-0,3769	-0,0177	0,0496	0,1304	0,8892	-0,4397	0,2561
V	1,8544	-0,6687	0,0766	0,0631	1,9217	0,4941	-0,2916	2,9768	0,1574	4,5885	0,0721	-0,1614	0,1529	-0,3365	0,4447	0,3325	0,7904	3,7804	-0,0581	1,0373
W	-0,2916	-0,9919	-0,5744	-0,543	-0,0581	-0,561	-0,8438	-0,1569	-0,7809	0,5075	-0,8034	-0,6911	-0,6148	-0,7091	-0,5026	-0,4936	-0,6507	-0,0581	-0,5789	-0,1255
Y	0,6826	-0,8393	0,2606	0,0676	0,8577	0,2786	-0,3544	0,8577	-0,2467	1,7107	-0,4352	-0,1344	0,2202	-0,3589	0,3774	0,0317	0,0631	0,9296	-0,2153	0,539

Table S7: Z-score normalization of amino amino acids residue-residue contacts frequencies in α/β class (117 PDB).

	A	C	D	E	F	G	H	I	K	L	M	N	P	Q	R	S	T	V	W	Y
A	1,2612	-0,7853	-0,2768	-0,1527	0,8829	0,3868	-0,2954	1,4162	-0,1713	2,8735	-0,3512	-0,2085	-0,2023	-0,4938	-0,0101	0,0085	0,2689	2,0673	-0,3078	0,5604
C	-0,6985	-0,2085	-0,9775	-0,8907	-0,8039	-0,8101	-0,9031	-0,5868	-0,8907	-0,3326	-1,0519	-1,0023	-0,7915	-0,9279	-0,9279	-0,8163	-0,7171	-0,4132	-1,0581	-0,593
D	-0,1775	-0,8783	-0,1837	-0,3202	-0,2148	0,0705	-0,2023	-0,3326	0,2938	0,1015	-0,8039	-0,1713	-0,4814	-0,5682	0,61	-0,1279	-0,0783	0,0085	-0,6116	0,1325
E	-0,252	-0,9899	-0,4442	-0,376	-0,1713	-0,1775	-0,6736	-0,1651	0,3124	0,3372	-0,8163	-0,3574	-0,314	-0,531	1,1619	-0,2272	-0,2706	0,1015	-0,7853	0,1697
F	0,7402	-0,5992	-0,1899	-0,0163	1,7138	0,1015	-0,3636	1,6952	-0,1899	3,4936	-0,3574	-0,3946	-0,0783	-0,4256	0,1449	0,1015	0,1139	1,5464	0,0581	0,8829
G	0,4178	-0,7791	0,2379	0,1387	0,3	0,7464	-0,438	0,486	-0,1093	1,1247	-0,655	-0,1837	-0,2768	-0,3946	0,1449	0,0891	0,1573	0,7278	-0,5434	0,7216
H	-0,5372	-0,9899	-0,345	-0,2085	-0,4318	-0,4132	-0,4194	-0,5062	-0,8783	0,0953	-0,8535	-0,6798	-0,6922	-0,7791	-0,6426	-0,4504	-0,469	-0,2706	-0,7605	-0,0783
I	1,2922	-0,7109	-0,2272	0,1697	1,689	0,393	-0,4442	3,5618	-0,1279	4,8889	0,0147	-0,3636	-0,1589	-0,4194	0,0829	0,3372	0,4054	2,9045	-0,1837	0,8395
K	-0,1527	-0,9589	0,4302	0,5108	-0,1899	-0,1527	-0,8225	0,1325	-0,407	0,548	-0,7853	-0,3078	-0,3574	-0,4008	-0,345	-0,1031	-0,1775	0,1263	-0,7915	0,1883
L	2,7433	-0,2892	0,2689	0,6534	3,2394	0,9573	0,0147	4,8269	0,5418	8,7151	0,5976	0,1821	0,5852	0,0829	1,0317	0,6968	1,1619	4,8703	0,4302	2,1045
M	-0,376	-1,0023	-0,7915	-0,6985	-0,1527	-0,6798	-0,9775	-0,1837	-0,9093	0,3434	-0,6985	-0,7853	-0,7171	-0,9217	-0,6736	-0,8411	-0,7233	-0,0287	-0,7915	-0,531
N	-0,4566	-1,0085	-0,3574	-0,3078	-0,2085	-0,1093	-0,5744	-0,4008	-0,3326	0,0085	-0,9713	-0,2892	-0,5868	-0,562	-0,1899	-0,2768	-0,3078	-0,1031	-0,655	-0,0163
P	-0,252	-0,9279	-0,5372	-0,3016	-0,252	-0,2458	-0,6426	-0,3078	-0,4876	0,5976	-0,8783	-0,593	-0,407	-0,7357	-0,2396	-0,407	-0,4442	-0,0907	-0,5496	0,2565
Q	-0,3388	-1,0581	-0,4814	-0,4628	-0,4256	-0,2334	-0,7419	-0,314	-0,5992	0,2565	-0,9093	-0,562	-0,4442	-0,6488	-0,3574	-0,5434	-0,3264	-0,1031	-0,7047	-0,1837
R	0,0767	-0,8039	0,7775	1,2612	0,0953	0,2317	-0,531	0,2131	-0,2768	1,2301	-0,7047	-0,1899	0,0147	-0,3822	0,2503	-0,0473	0,0891	0,5728	-0,438	0,6224
S	-0,1155	-0,7109	-0,0535	-0,2458	0,0643	-0,0473	-0,4504	0,1697	-0,3202	0,6286	-0,7853	-0,345	-0,3016	-0,4442	-0,0535	-0,0101	-0,1899	0,3682	-0,6922	0,2007
T	0,4178	-0,7481	-0,2023	-0,0845	0,1201	0,4302	-0,5186	0,672	0,0023	1,2612	-0,7233	-0,2768	-0,1837	-0,4628	0,0333	-0,1589	-0,0287	0,7526	-0,6364	0,2814
V	1,8317	-0,5372	-0,1899	0,3496	1,5464	0,7154	-0,314	2,6937	0,0643	5,1308	-0,0473	-0,1527	0,3062	-0,2272	0,3124	0,362	0,6348	3,7541	-0,2396	1,317
W	-0,4256	-0,9899	-0,624	-0,7295	-0,2706	-0,3264	-0,9837	-0,2892	-0,8411	0,4984	-0,8783	-0,8163	-0,5	-0,8597	-0,593	-0,6302	-0,7295	-0,1961	-0,7977	-0,345
Y	0,6534	-0,6985	0,4178	-0,0101	1,1185	0,5232	-0,376	1,0689	0,0271	2,0301	-0,3636	-0,2458	0,2752	-0,3884	0,5108	-0,0039	0,2752	1,0813	-0,3636	0,6968

Table S8: Z-score normalization of amino amino acids residue-residue contacts frequencies in α+β class (126 PDB).

4. Quantitative analysis of residue-specific contact frequencies across four structural classes of hydrolase proteins

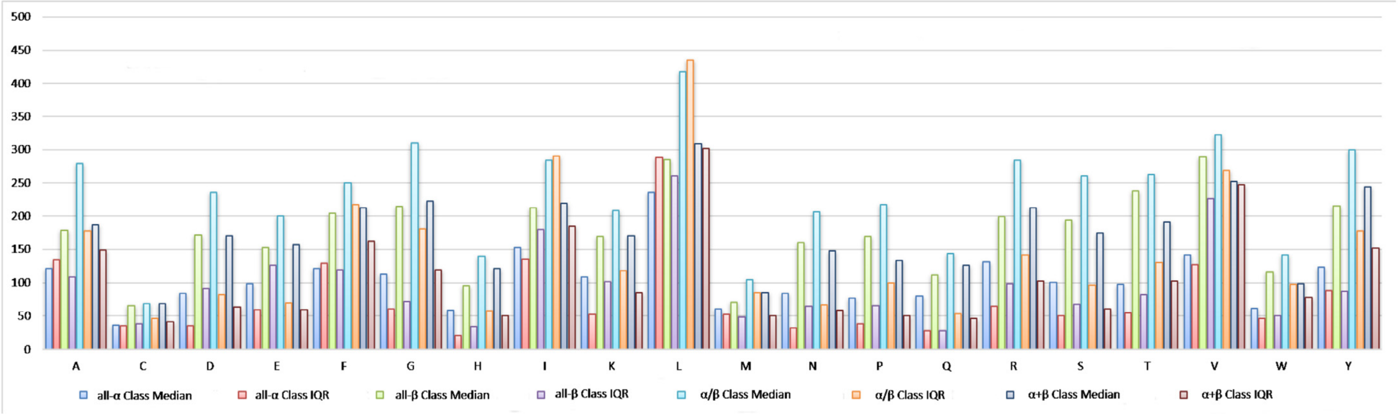

Figure S2: Median distribution and IQR overlap statistics of amino acid contact profiles under universal space-packing constraints. Bars represent the median contact frequencies and corresponding interquartile ranges (IQR) for the 20 standard amino acids, desaggregated into all-α, all-β, α/β, α+β structural folds of hydrolase superfamily.

5. Pearson correlation of raw and normalized intra-class residue contact frequencies in structural classes of hydrolase proteins

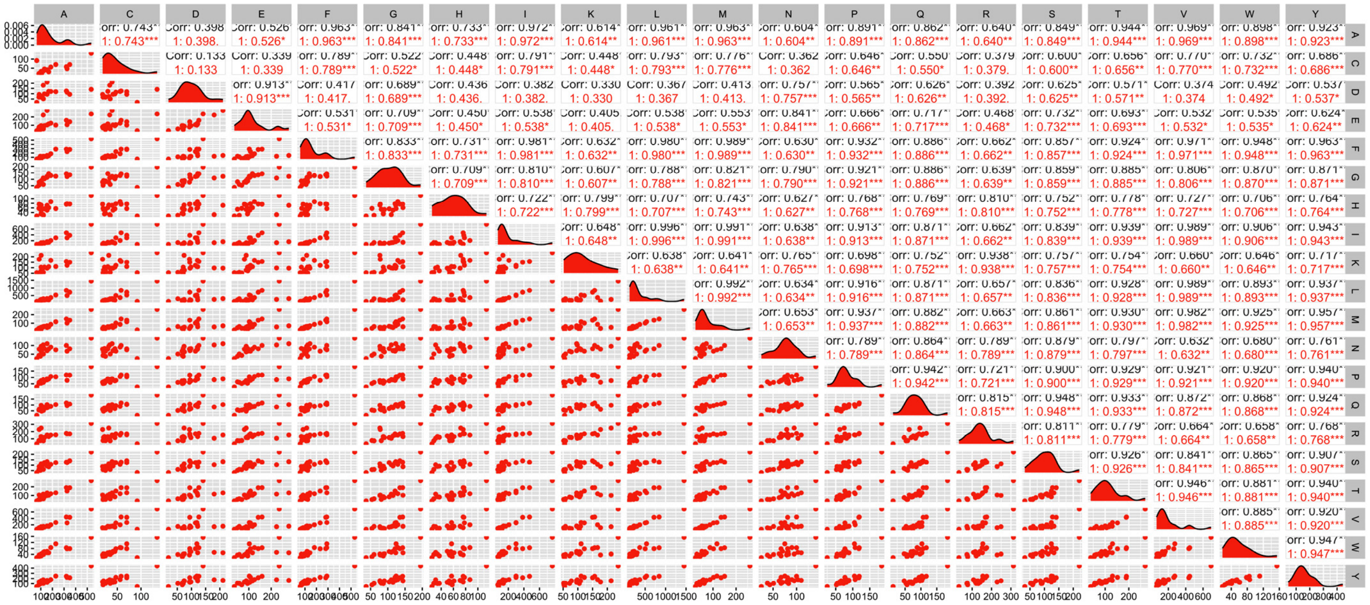

Figure S3: Intra-Class Stability Correlogram. The plot represents the PCCs of raw, and normalized RRCF for structural class all-α (118 PDB) of hydrolase proteins. The diagonal displays the PCC of identical raw and normalized RRCF. The lower panel displays scatter plots and smoothing splines for for each pair of variables; the upper panel gives the corresponding PCC, with text size proportional to its absolute value. In the lower panel, smoothing is performed through locally weighted polynomial regressions. In the upper panel, each correlation coefficient is tested against the null hypothesis and the resulting p-value is symbolically encoded at the levels of 0.05 (*), 0.01 (**), and 0.001 (***).

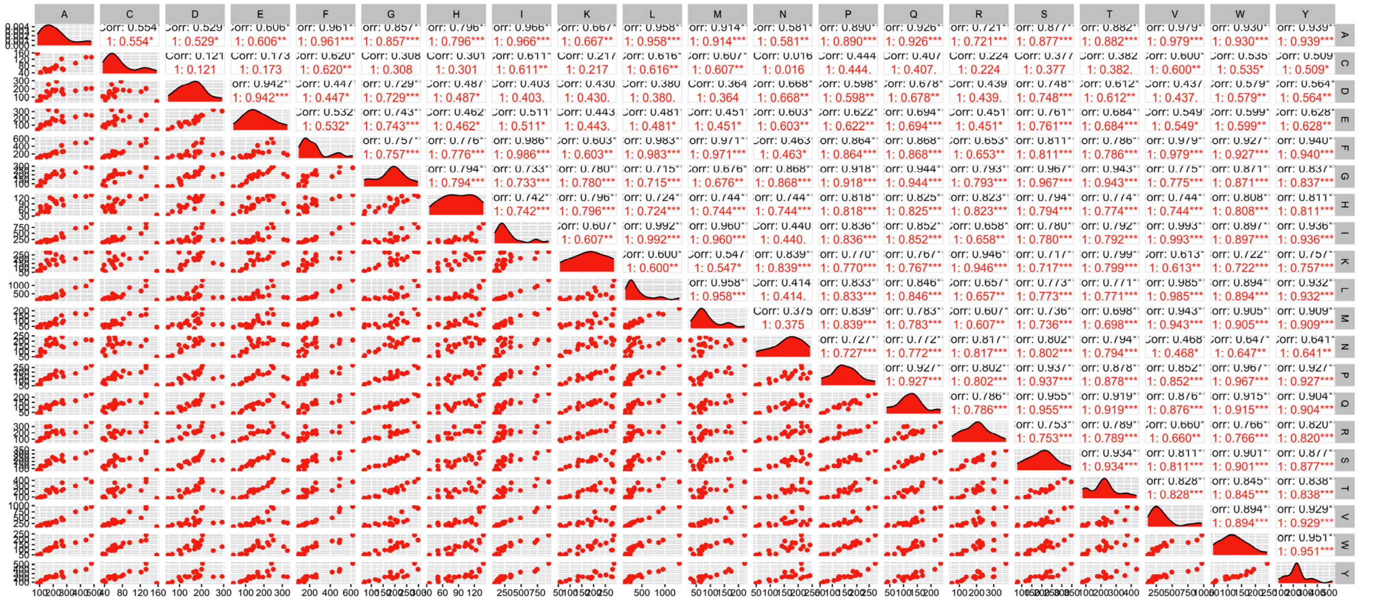

Figure S4: Intra-Class Stability Correlogram. The plot represents the PCCs of raw, and normalized RRCF of the structural class all-β (114 PDB) of hydrolase proteins. The diagonal displays the PCC of identical raw and normalized RRCF. The lower panel displays scatter plots and smoothing splines for each pair of variables; the upper panel gives the corresponding PCC, with text size proportional to its absolute value. In the lower panel, smoothing is performed through locally weighted polynomial regressions. In the upper panel, each correlation coefficient is tested against the null hypothesis and the resulting p-value is symbolically encoded at the levels of 0.05 (*), 0.01 (**), and 0.001 (***).

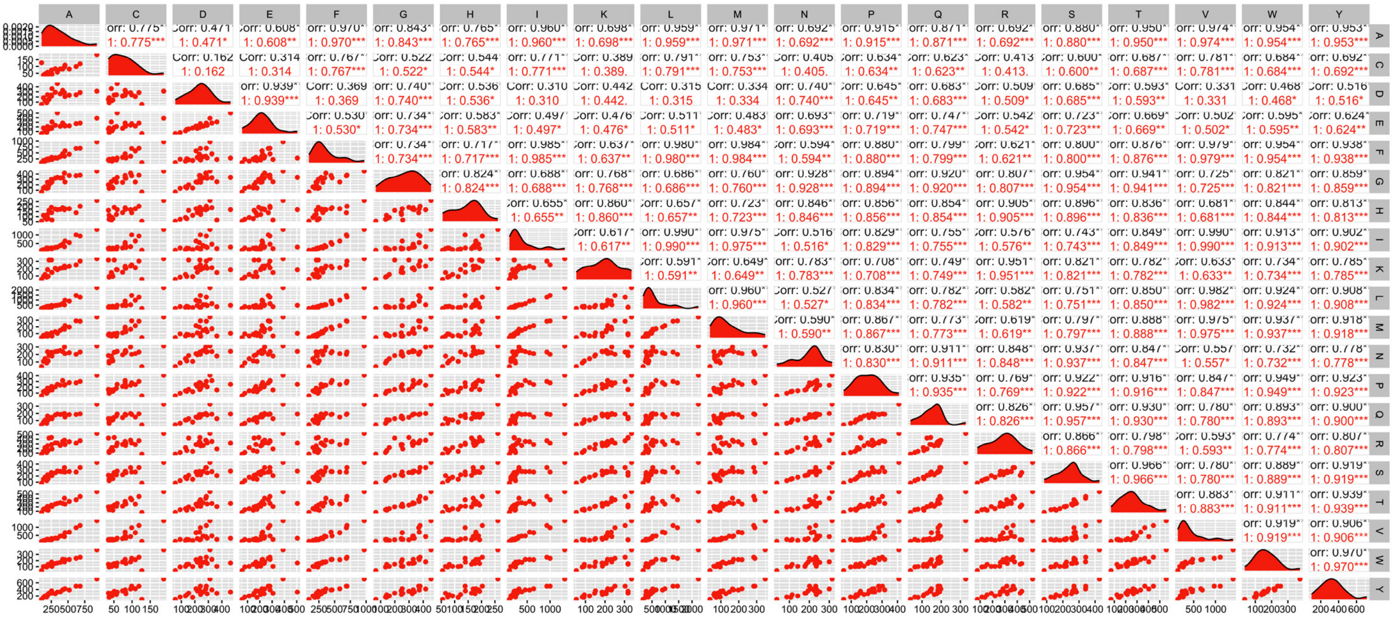

Figure S5: Intra-Class Stability Correlogram. The plot represents the PCCs of raw and normalized RRCF for structural class α/β (117 PDB) of hydrolase proteins. The diagonal displays the PCC of identical raw and normalized RRCF. The lower panel displays scatter plots and smoothing splines for each pair of variables; the upper panel gives the corresponding PCC, with text size proportional to its absolute value. In the lower panel, smoothing is performed through locally weighted polynomial regressions. In the upper panel, each correlation coefficient is tested against the null hypothesis and the resulting p-value is symbolically encoded at the levels of 0.05 (*), 0.01 (**), and 0.001 (***).

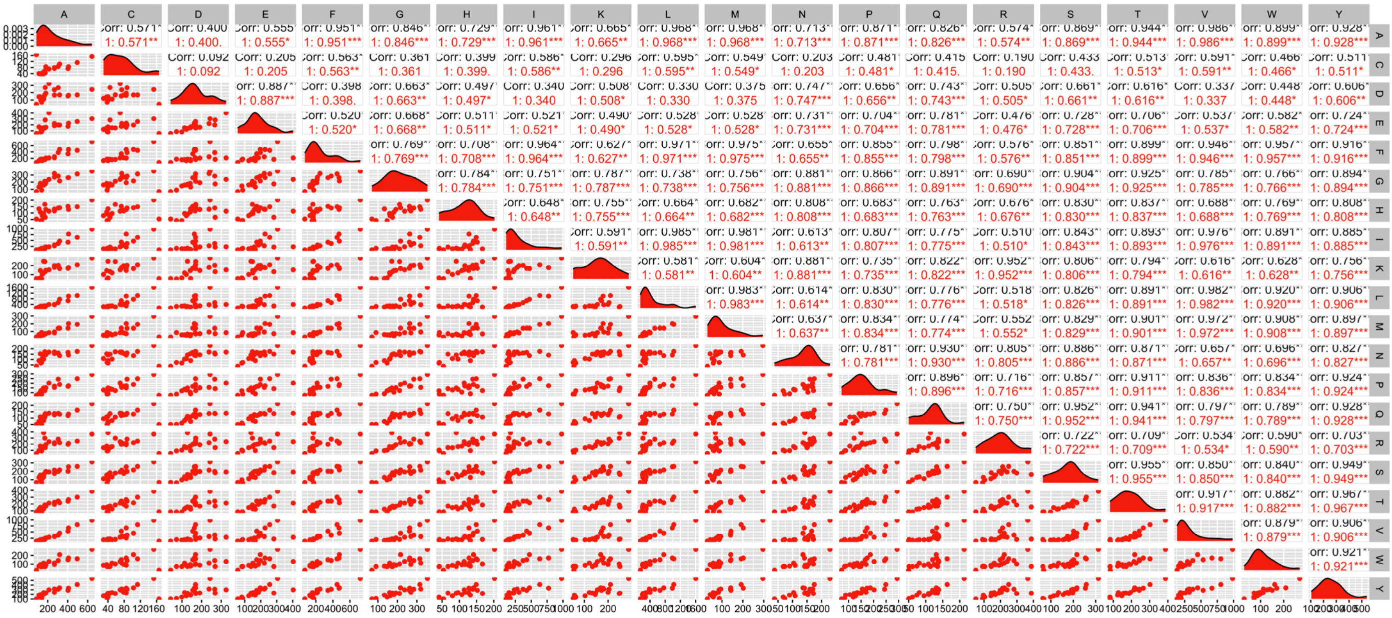

Figure S6: Intra-Class Stability Correlogram. The plot represents the PCCs of raw and normalized RRCF for structural class $\alpha+\beta$ (126 PDB) of hydrolase proteins. The diagonal displays the PCC of identical raw and normalized RRCF. The lower panel displays scatter plots and smoothing splines for each pair of variables; the upper panel gives the corresponding PCC, with text size proportional to its absolute value. In the lower panel, smoothing is performed through locally weighted polynomial regressions. In the upper panel, each correlation coefficient is tested against the null hypothesis and the resulting p-value is symbolically encoded at the levels of 0.05 (*), 0.01 (**), and 0.001 (***).

6. Pearson correlation of raw and normalized residue-residue contacts frequencies between structural classes of hydrolase proteins

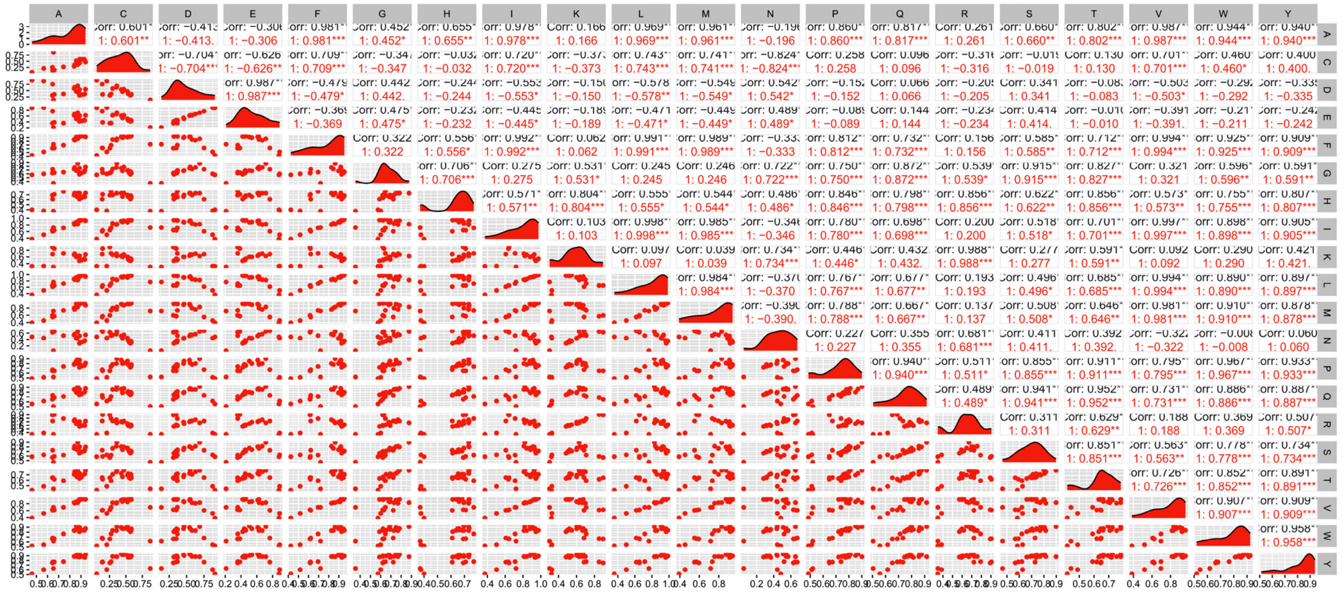

Figure S7: Inter-Class Stability Correlogram.The plot represents the PCCs of raw and normalized RRCF between the structural classes all-α (118 PDB) and all-β (114 PDB) of hydrolase proteins. The diagonal displays the PCC of identical raw and normalized RRCF. The lower panel displays scatter plots and smoothing splines for each pair of variables; the upper panel gives the corresponding PCC, with text size proportional to its absolute value. In the lower panel, smoothing is performed through locally weighted polynomial regressions. In the upper panel, each correlation coefficient is tested against the null hypothesis and the resulting p-value is symbolically encoded at the levels of 0.05 (*), 0.01 (**), and 0.001 (***).

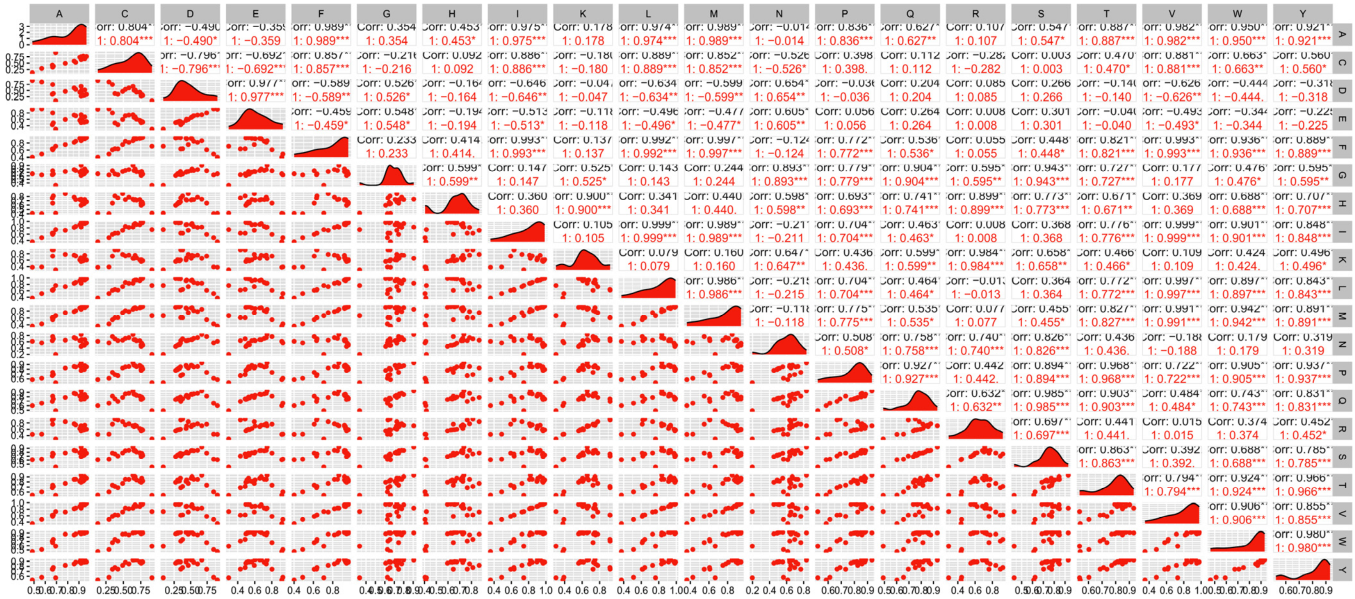

Figure S8: Inter-Class Stability Correlogram. The plot represents PCCs of raw and normalized RRCF between the structural classes all-α (118 PDB) and α/β (117 PDB) of hydrolase proteins. The diagonal displays the PCC of identical raw and normalized RRCF. The lower panel displays scatter plots and smoothing splines for each pair of variables; the upper panel gives the corresponding PCC, with text size proportional to its absolute value. In the lower panel, smoothing is performed through locally weighted polynomial regressions. In the upper panel, the correlation coefficient is tested against the null hypothesis and the resulting p-value is symbolically encoded at the levels of 0.05 (*), 0.01 (**), and 0.001 (***).

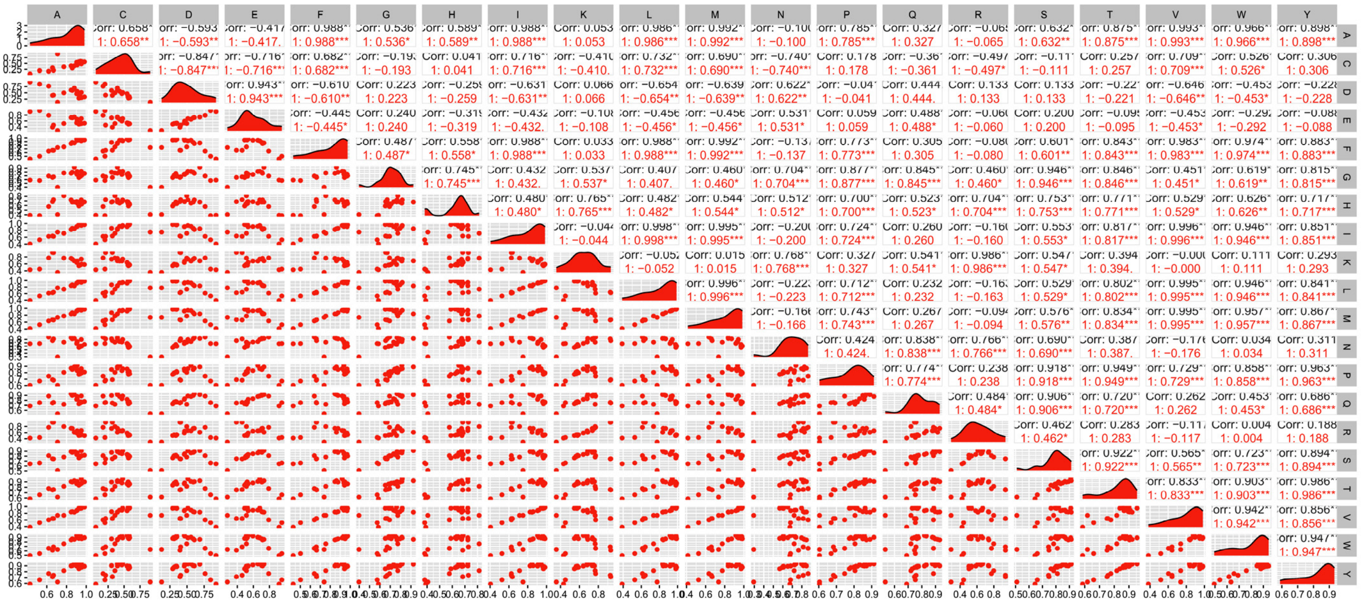

Figure S9: Inter-Class Stability Correlogram. The plot represents PCCs of raw and normalized RRCF between the structural classes of all-α (118 PDB) and α+β (126 PDB) of hydrolase proteins. The diagonal displays the PCC of identical raw and normalized RRCF. The lower panel displays scatter plots and smoothing splines for each pair of variables; the upper panel gives the corresponding PCC, with text size proportional to its absolute value. In the lower panel, smoothing is performed through locally weighted polynomial regressions. In the upper panel, the correlation coefficient is tested against the null hypothesis and the resulting p-value is symbolically encoded at the levels of 0.05 (*), 0.01 (**), and 0.001 (***).

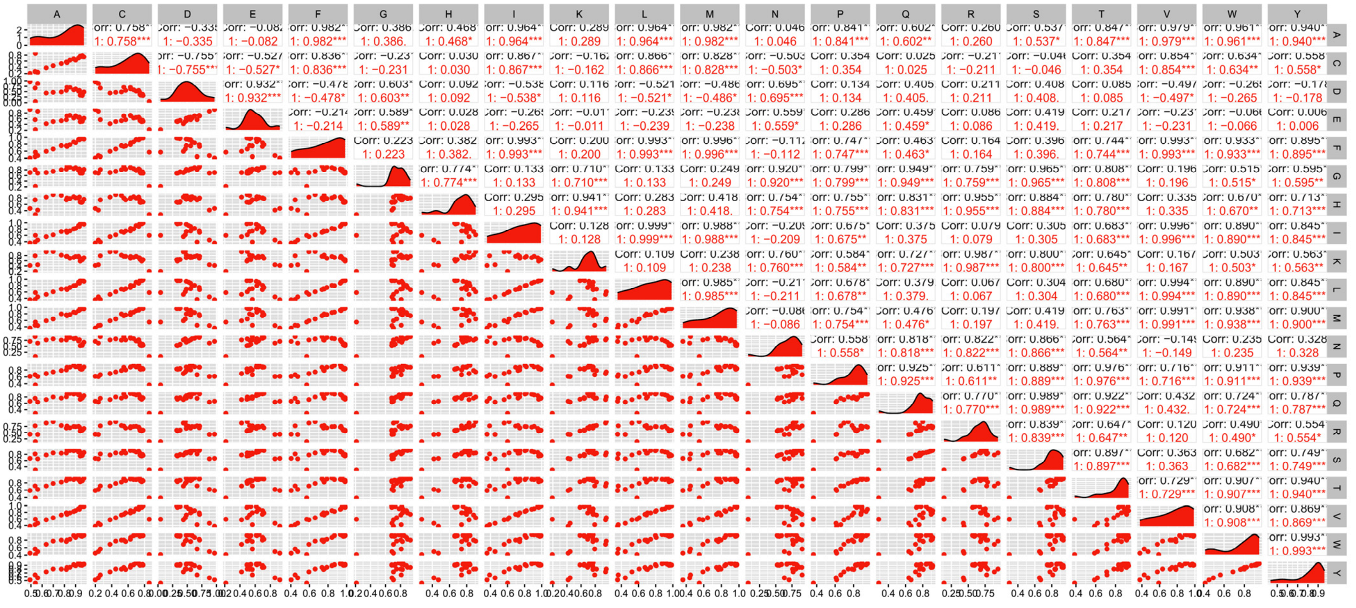

Figure S10: Inter-Class Stability Correlogram. The plot represents PCCs of raw and normalized RRCF between the structural classes all-β (114 PDB) and α/β (117 PDB) of hydrolase proteins. The diagonal displays the PCC of identical raw and normalized RRCF. The lower panel displays scatter plots and smoothing splines for each pair of variables; the upper panel gives the corresponding PCC, with text size proportional to its absolute value. In the lower panel, smoothing is performed through locally weighted polynomial regressions. In the upper panel, each correlation coefficient is tested against the null hypothesis and the resulting p-value is symbolically encoded at the levels of 0.05 (*), 0.01 (**), and 0.001 (***).

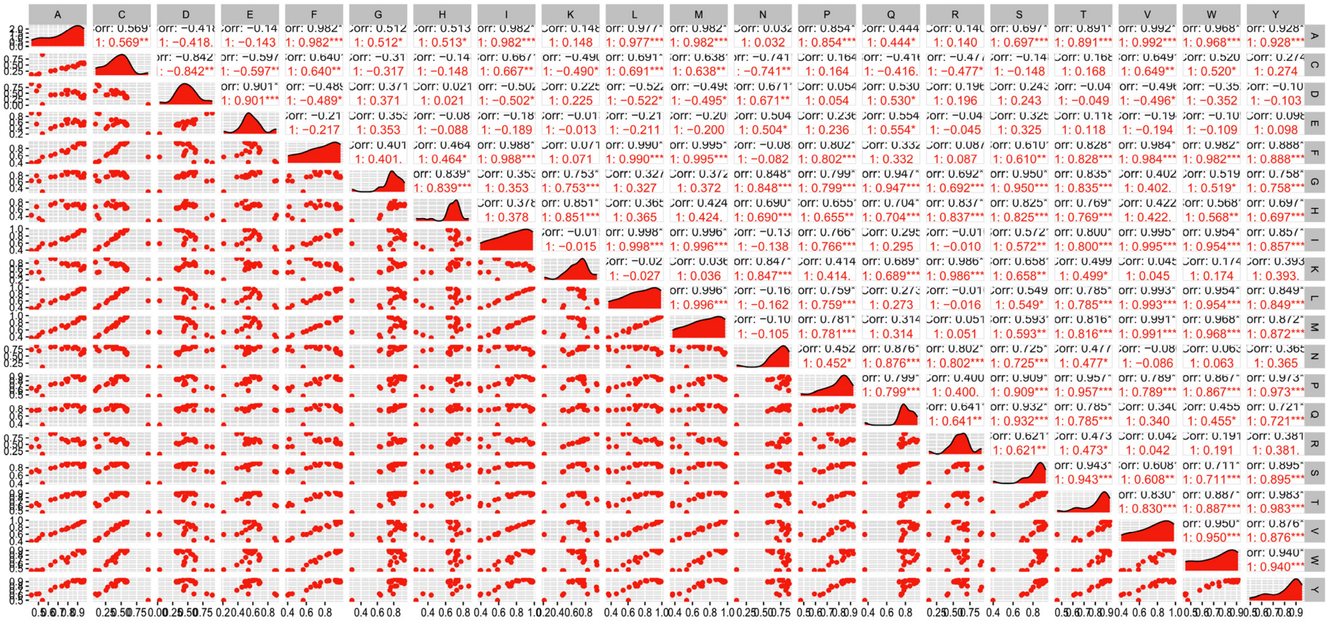

Figure S11: Inter-Class Stability Correlogram. The plot represents PCCs of raw and normalized RRCF between the structural classes all-β (114 PDB) and α+β (126 PDB) of hydrolase proteins. The diagonal displays the PCC of identical raw and normalized RRCF. The lower panel displays scatter plots and smoothing splines for each pair of variables; the upper panel gives the corresponding PCC, with text size proportional to its absolute value. In the lower panel, smoothing is performed through locally weighted polynomial regressions. In the upper panel, the correlation coefficient is tested against the null hypothesis and the resulting p-value is symbolically encoded at the levels of 0.05 (*), 0.01 (**), and 0.001 (***).

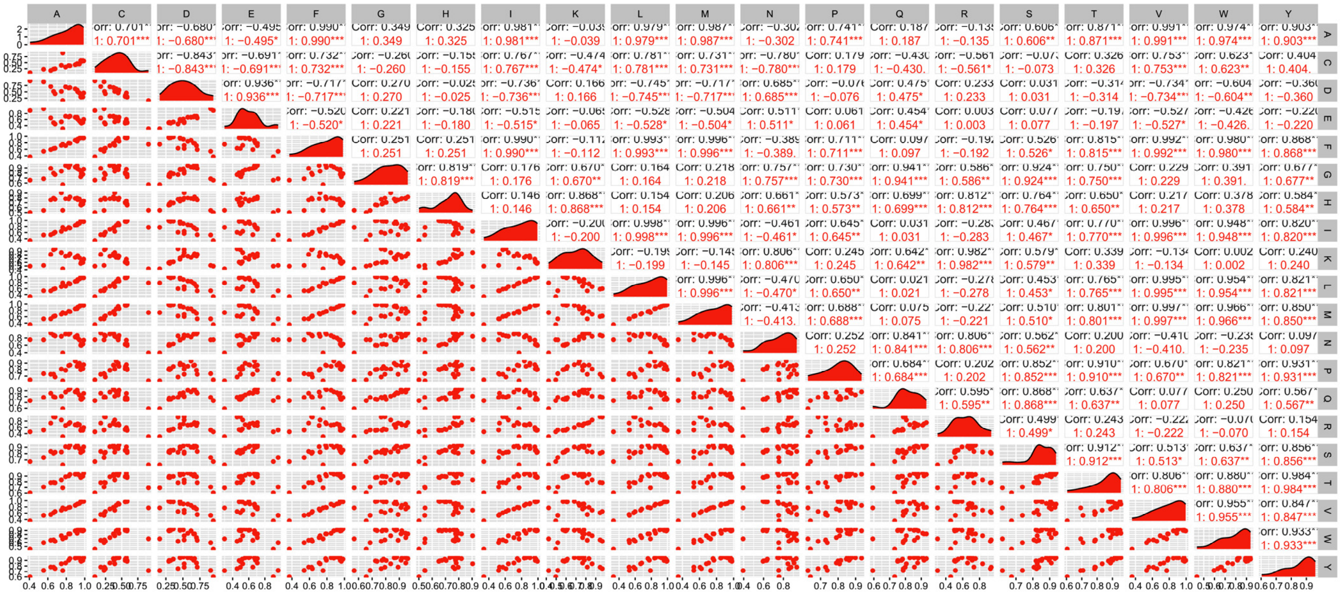

Figure S12: Inter-Class Stability Correlogram. The plot represents PCCs of raw and normalized RRCF between the structural classes of α/β (117 PDB) and α+β (126 PDB) of hydrolase proteins. The diagonal displays the PCC of identical raw and normalized RRCF. The lower panel displays scatter plots and smoothing splines for each pair of variables; the upper panel gives the corresponding PCC, with text size proportional to its absolute value. In the lower panel, smoothing is performed through locally weighted polynomial regressions. In the upper panel, each correlation coefficient is tested against the null hypothesis and the resulting p-value is symbolically encoded at the levels of 0.05 (*), 0.01 (**), and 0.001 (***).
